# Publisher Correction: Polymorphic design of DNA origami structures through mechanical control of modular components

**DOI:** 10.1038/s41467-018-02948-z

**Published:** 2018-02-07

**Authors:** Chanseok Lee, Jae Young Lee, Do-Nyun Kim

**Affiliations:** 1 Department of Mechanical and Aerospace Engineering, Seoul National University, 301-dong 116-ho, 1 Gwanak-ro, Gwanak-gu, Seoul 08826 Korea; 2 Institute of Advanced Machines and Design, Seoul National University, 313-dong 320-ho, 1 Gwanak-ro, Gwanak-gu, Seoul 08826 Korea

Correction to: *Nature Communications* 10.1038/s41467-017-02127-6, published online 12 December 2017

The originally published version of this Article contained an error in Figure 5. In panel f, the right *y*-axis ‘Strain energy (kbT)’ was labelled ‘Probability’ and the left *y*-axis ‘Probability’ was labelled ‘Strain energy (kbT)’. This error has now been corrected in both the PDF and HTML versions of the Article.

