# Supplementary material for: Polymorphic design of DNA origami structures through mechanical control of modular components
Source: Nat Commun. 2017 Dec 12;8:2067. doi: 10.1038/s41467-017-02127-6 (PMC5727162; doi:10.1038/s41467-017-02127-6)
Supplement: Supplementary file 1 — Supplementary Information [file 41467_2017_2127_MOESM1_ESM.pdf]

Supplementary Methods

Finite element analysis (CanDo)

In CanDo, double-stranded DNA (dsDNA) is modeled as two-node beam elements in which each node represents a basepair. The beam has proper geometry (diameter of 2.25 nm and axial rise of 0.34 nm per each base, and helicity of 10.5 basepair (bp) per turn) and mechanical properties (axial rigidity of 1,100 pN, bending rigidity of 230 pN nm^2, and torsional rigidity of 460 pN nm^2), which correspond to experimentally validated values of dsDNA. Also, a Holliday junction (crossover) is modeled as two rigid beams. We made some modification in the modeling of DNA origami structures compared with original CanDo[1]. At first, DNA single-strand breaks, known as nicks, are modeled having the same bending and torsional stiffness as dsDNA, since the effect of nick stiffness is negligible in terms of predicting an equilibrium included angle of the structures designed here. Also, we adopted wormlike-chain (WLC) model to describe a single-stranded DNA (ssDNA) strand as an entropic spring, instead of modified freely jointed chain (mJFC) model used in previous CanDo. It is mainly due to enhancing the accuracy of predicting tensional force of the short ssDNA at the hinge module.

WLC force of ssDNA can be expressed as[2]

$$F_{\mathrm{WLC}} = \frac{k_{\mathrm{B}}T}{L_{\mathrm{p}}}\left[\frac{x}{L_{\mathrm{c}}} + \frac{1}{4(1-x/L_{\mathrm{c}})^2} - \frac{1}{4}\right] \quad (1)$$

where k_{B} denotes the Boltzmann constant, T is the temperature, L_{p} is the persistence length of ssDNA, x is the extension (end-to-end distance), L_{c} is the contour length, respectively. We set parameters as k_{B} = 4.1124 pN nm, T = 298 K, L_{p} = 2 nm, and L_{c} = 0.65 × number of base.

For finite element analysis, equation (1) is converted to axial stress-strain relationship implemented as

$$\sigma_{\mathrm{WLC}} = \frac{k_{\mathrm{B}}T}{L_{\mathrm{p}}}\left[\frac{R_0(1+\varepsilon)}{L_{\mathrm{C}}} + \frac{1}{4(1-R_0(1+\varepsilon)/L_{\mathrm{c}})^2} - \frac{1}{4}\right] \quad (2)$$

where R_0 is the initial length and ε is the axial strain, respectively.

Lastly, in case of modeling a hinge module consisting of only ssDNA strands, a certain amount of bases were modeled as dsDNA beams in order to give proper elastic stiffness. A more detailed and quantitative model of the hinge containing ssDNA helices may be required to increase the accuracy of CanDo shape prediction, which will be the subject of our future work.

Calculation of the hinge stiffness and strain energy of the structure

We used a simple toy model to calculate the effective bending stiffness of the flexible hinge. The hinge module is modeled as a linear torsional spring whose spring constant is κ and equilibrium position is straight. The ssDNA adjuster strand is modeled as a nonlinear axial spring whose force-displacement relationship is followed by WLC model as described in the equation (1). By adopting assumptions that all parts of the structure except for the hinge and the adjuster strand are rigid and DNA origami structures measured in the experiment were in the force-equilibrium state, we can use the average included angle to calculate the effective bending stiffness of the hinge, κ. It can be modeled as

$$\kappa(\pi - 2\theta) = RF_{\mathrm{WLC}} \cos\theta \qquad (3)$$

where θ denotes the half of the average included angle, R is the moment arm length defined as the length between the connecting point of the adjuster strand and the center of the hinge module (assumed as 85.7 nm), and F_{WLC} is the tensional force induced from the ssDNA adjuster, calculated from the equation (1).

From the effective bending stiffness, the total strain energy of the structure can be estimated by assuming that it is the summation of the strain energy of the hinge and ssDNA adjuster, respectively.

$$E_{\mathrm{tot}} = E_{\mathrm{h}} + E_{\mathrm{s}} = \frac{1}{2}\kappa(\pi - 2\theta)^2 + \int_0^l \frac{k_{\mathrm{B}}T}{L_{\mathrm{p}}}\left[\frac{x}{L_{\mathrm{c}}} + \frac{1}{4(1-x/L_{\mathrm{c}})^2} - \frac{1}{4}\right] dx \qquad (4)$$

where l is the end-to-end distance of the adjuster.

Principal component analysis of MD data

Principal component analysis (PCA) was performed to characterize the mechanical rigidities from the MD simulation using configurations of phosphorus atoms of each structure in equilibrium (5,000 snapshots for 20 ns). Let $\mathbf{x}(t)$ be the atomic coordinates from MD simulation at equilibrium with a dimension of 1 × 3N where N is a total number of phosphorus atoms at a saved frame. The covariance matrix is determined as[3]

$$\boldsymbol{\sigma} = \left\langle \left(\mathbf{x}(t) - \langle \mathbf{x}(t) \rangle\right) \otimes \left(\mathbf{x}(t) - \langle \mathbf{x}(t) \rangle\right) \right\rangle \qquad (5)$$

where a symbol, $\otimes$ indicates the tensor product, and an angle bracket is the vector of average. Then square-root-mass-weight matrix, $\boldsymbol{\Sigma}$ is obtained as

$$\boldsymbol{\Sigma} = \mathbf{M}^{1/2}\boldsymbol{\sigma}\mathbf{M}^{1/2} \qquad (6)$$

where a mass matrix, $\mathbf{M}$ is a diagonal matrix with elements of the atomic weight of phosphorus atom. Then we can obtain the eigenvalues, λ_n from the diagonalization of the square-root-mass-weight matrix. The quasi-harmonic frequencies, ω_n of the nth mode, can be calculated as

$$\omega_n = \sqrt{\frac{k_\mathrm{B}T}{\lambda_n}} \tag{7}$$

Elastic bending and stretching rigidities of DNA structures are approximately calculated using the quasi-harmonic frequencies based on the dynamic Euler-Bernoulli beam model as[4]

$$EI_n = \frac{M\omega_n{}^2L^3}{\left(\beta_n L\right)^4} \qquad EA_n = \frac{M\,\omega_n{}^2L}{n^2\pi^2} \tag{8}$$

where EI_n and EA_n are respectively bending and stretching rigidities of the nth mode for the boundary conditions of free-free ends, M and L are the total mass and the axial length of a DNA structure, and $\beta_n L$ is a known constant determined from boundary conditions, respectively ($\beta_1 L = 4.733$).

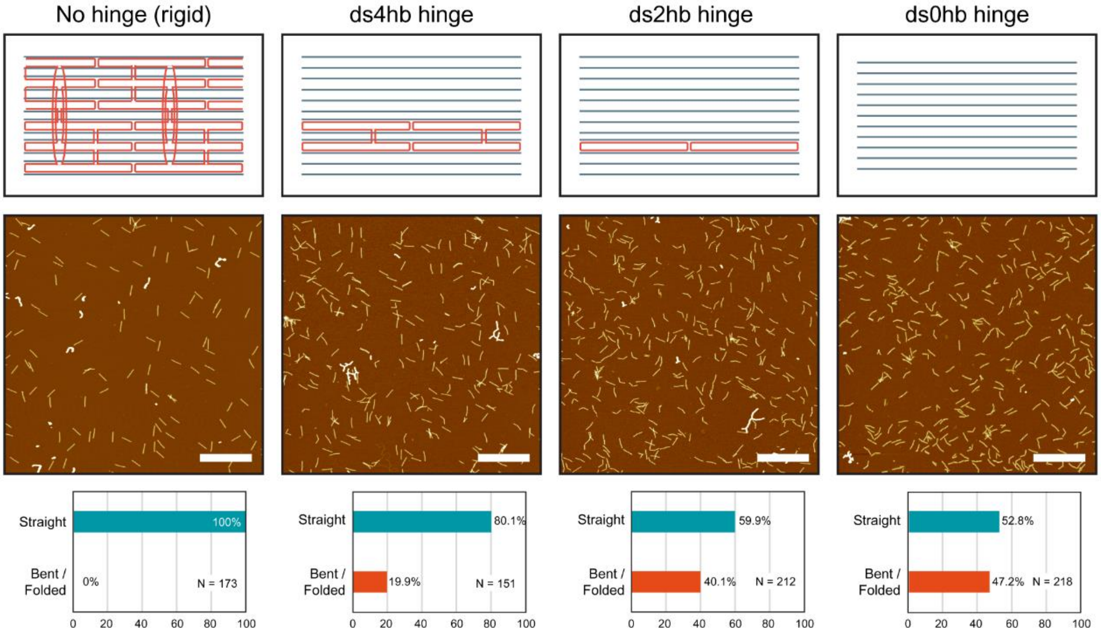

Supplementary Figure 1. The effect of hinge stiffness without an adjuster component. The percentages of straight structures and bent or folded structures are presented below. Results show that the portion of bent and folded structures increased as the hinge became more flexible, but at least more than half of the structures remained in straight configurations. Average included angle of ds0hb hinge design was approximately 155° including straight structures. Scale bars: 1 μm.

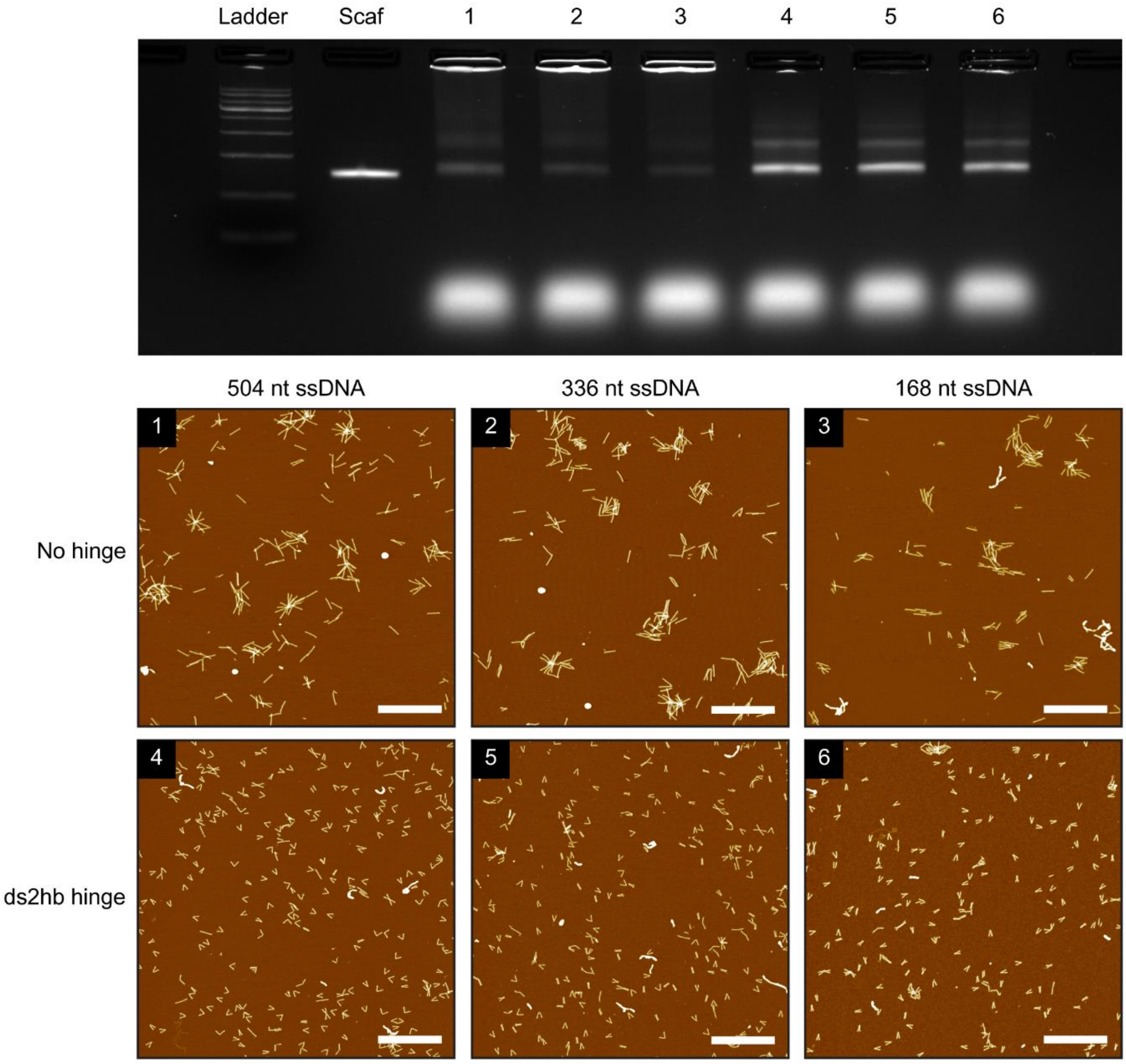

Supplementary Figure 2. Agarose gel electrophoresis and AFM images showing the effect of the hinge with different length of ssDNA adjuster strands. Structures without hinge (cases 1 to 3) showed drastically decreased monomer folding yield compared with that of structures with a ds2hb hinge (cases 4 to 6). Scale bars: 1 μm.

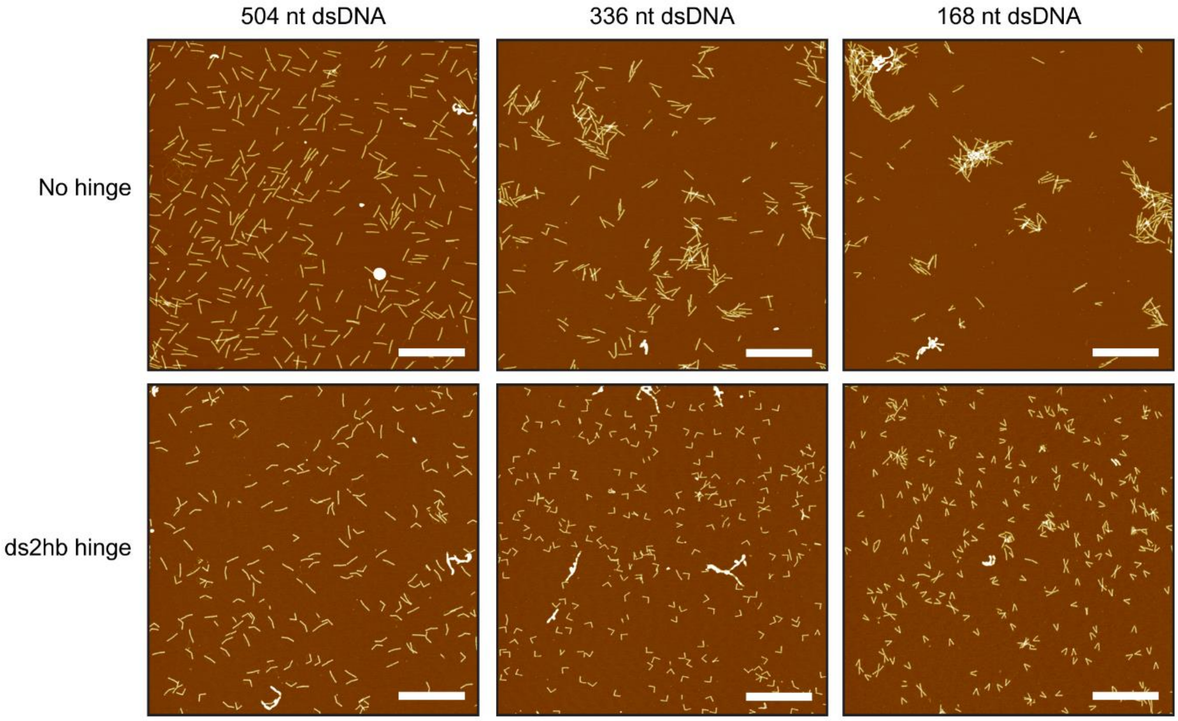

Supplementary Figure 3. AFM images showing the effect of the hinge with different length of dsDNA adjuster strands. Here, no hinge structure with the 504-nt-long dsDNA adjuster strand is same as the reference structure in the main text, and it mostly showed straight conformation with high monomer yield. No hinge structures with shorter dsDNA adjuster strand showed decreased monomer folding yield same as the ssDNA adjuster cases. Scale bars: 1 μm.

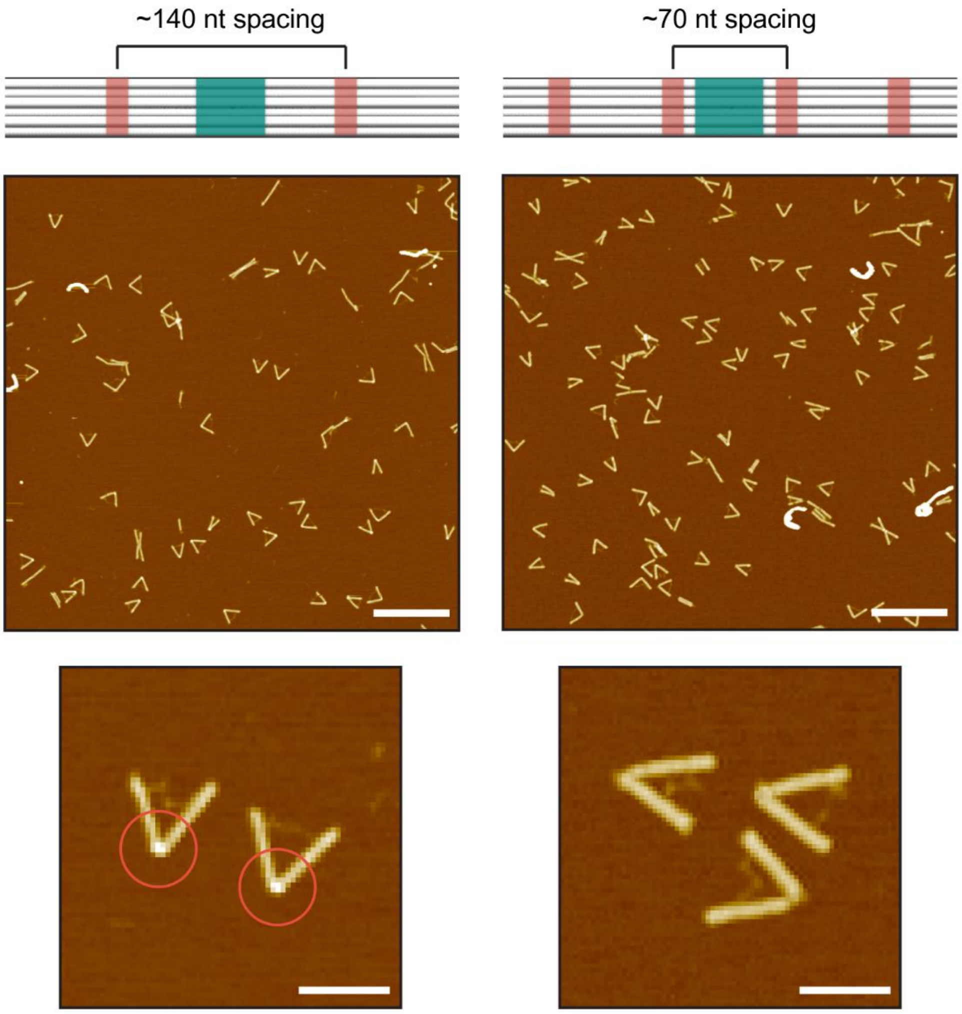

Supplementary Figure 4. Effect of the scaffold crossover seam spacing while remaining the length of the hinge region as 42 nt (green region). Structures with longer crossover spacing tended to show increased amount of overlapping at the vertex, which may be attributed to the increased instability near the hinge region. Scale bars in large-area images: 500 nm. Scale bars in cropped images: 100 nm.

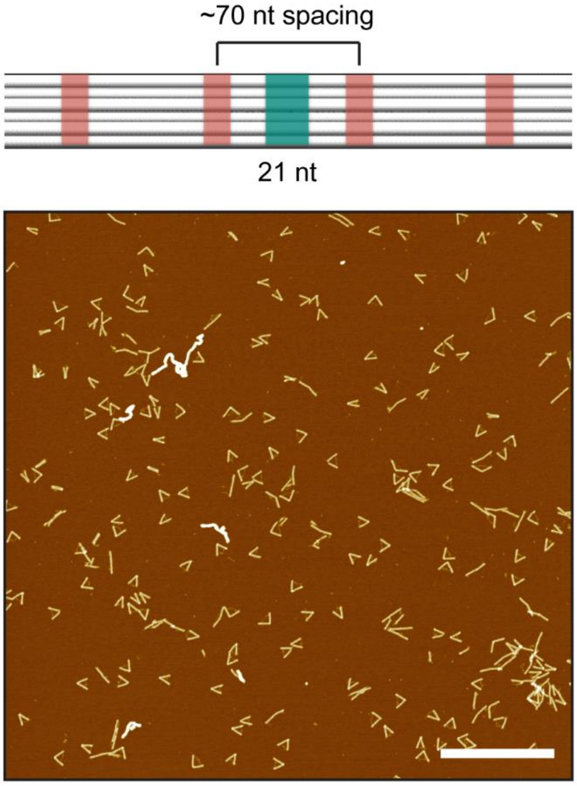

Supplementary Figure 5. AFM image of the 21-nt-long ds0hb hinge structure with the 504-nt-long ssDNA adjuster strand. Scale bar: 1 µm.

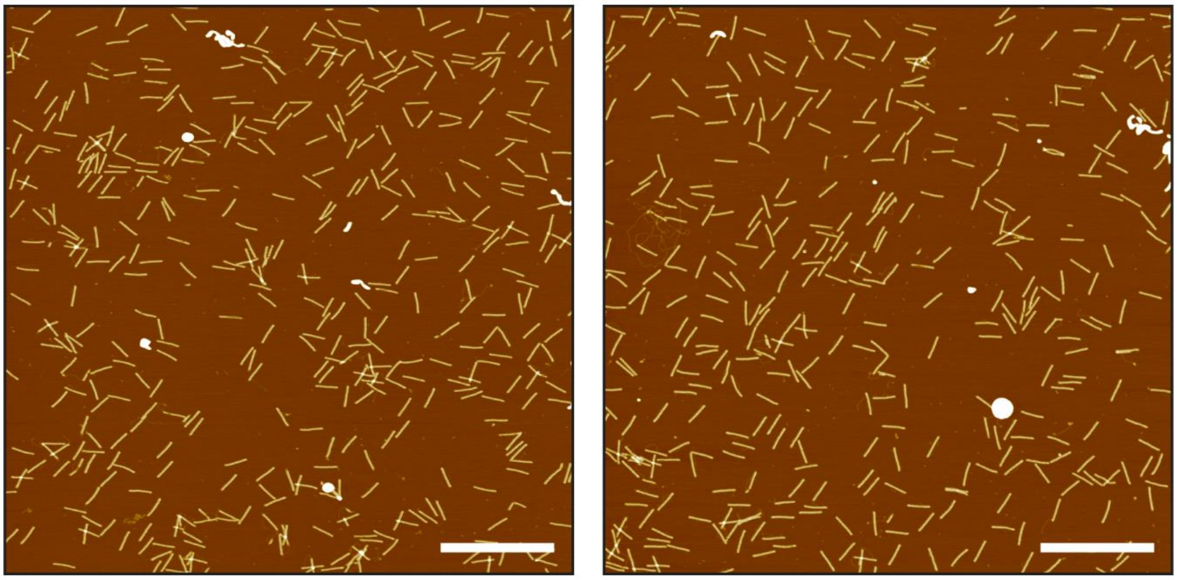

Supplementary Figure 6. AFM image of the reference structure shown in Fig. 2. Scale bars: 1 μm.

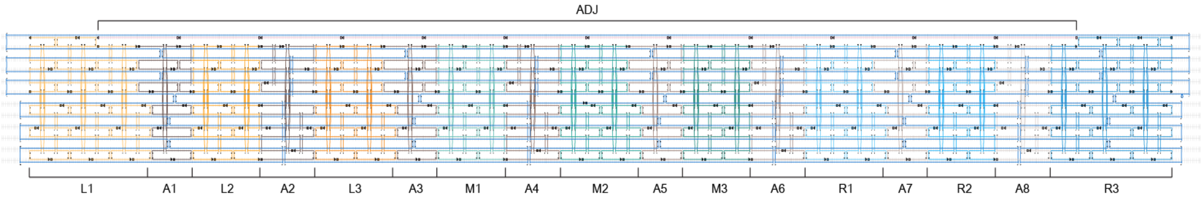

Supplementary Figure 7. Scaffold and staple layout of the reference design. The design of scaffold pathway and staple connectivity was done using the caDNAno software[5]. The reference shape has nine module regions (L1 to L3, M1 to M3, and R1 to R3), eight seam regions (A1 to A8), and adjuster strut staples (ADJ). Scaffold crossover planes were located in A1 to A8 and the center of R3 region. Detailed information of the reference staple set is shown in Supplementary Table 1.

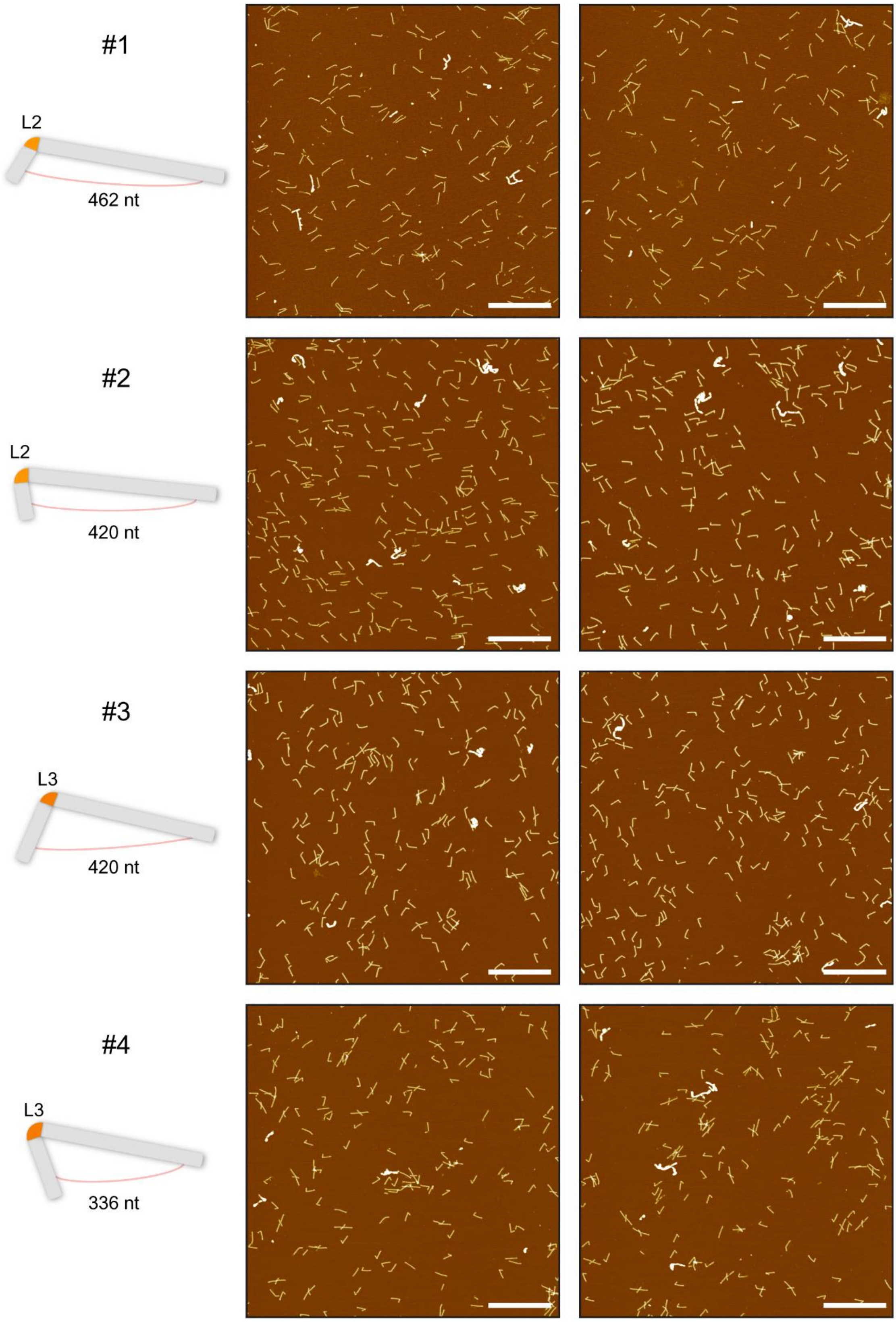

Supplementary Figure 8. AFM images of the structures #1 to #4 shown in Fig. 2. Scale bars: 1 µm.

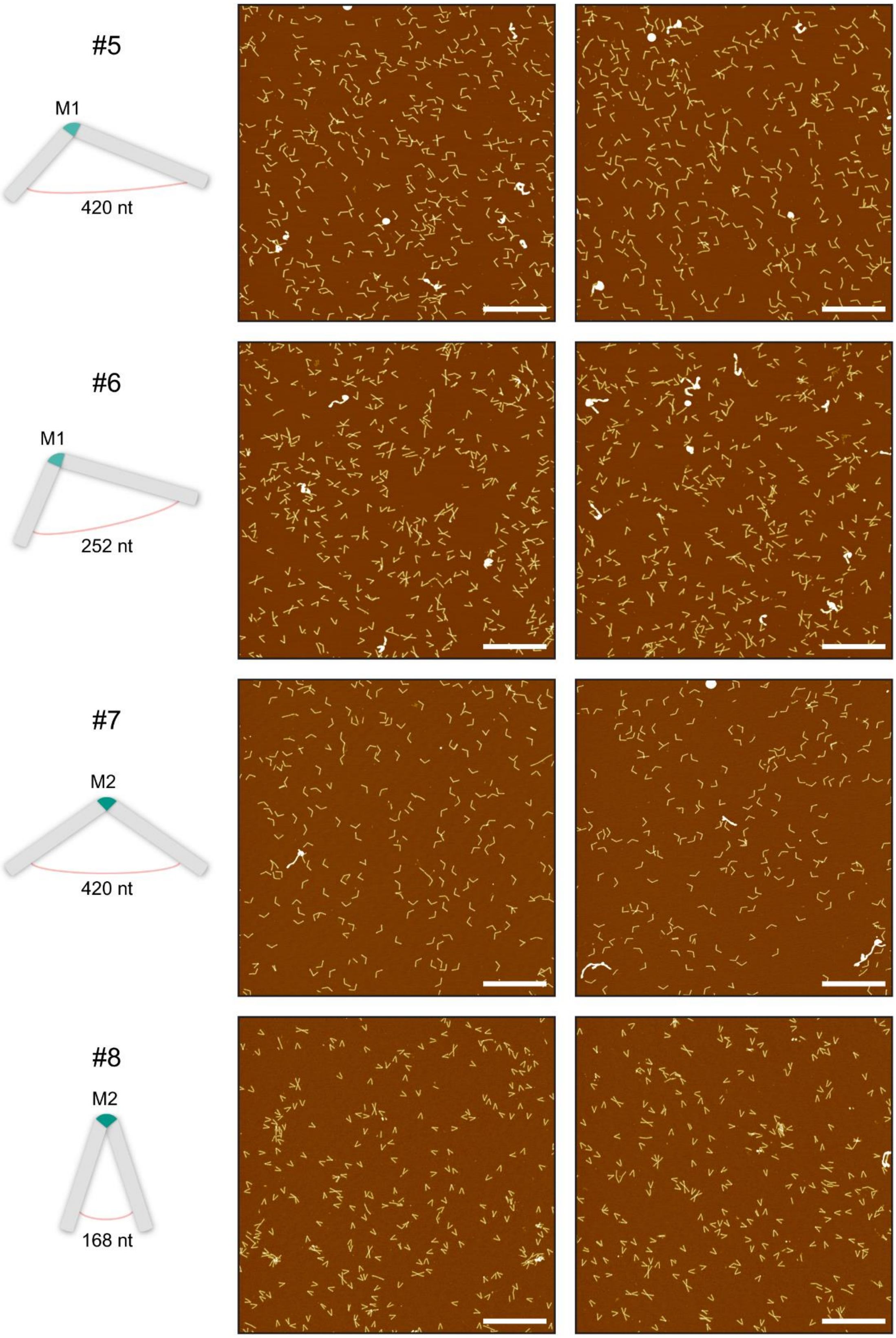

Supplementary Figure 9. **AFM images of the structures #5 to #8 shown in Fig. 2.** Scale bars: 1 μm.

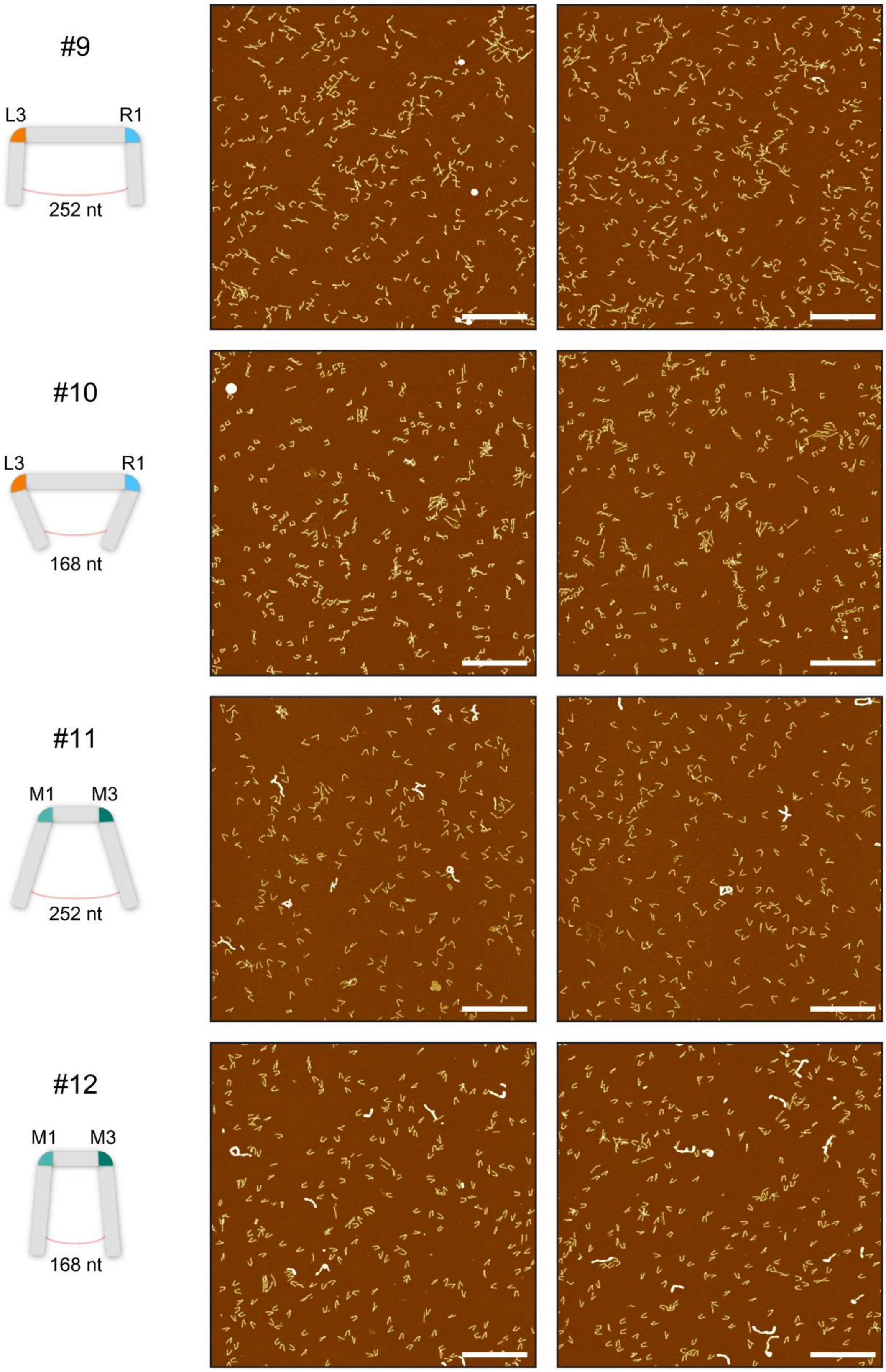

Supplementary Figure 10. AFM images of the structures #9 to #12 shown in Fig. 2. Scale bars: 1 μm.

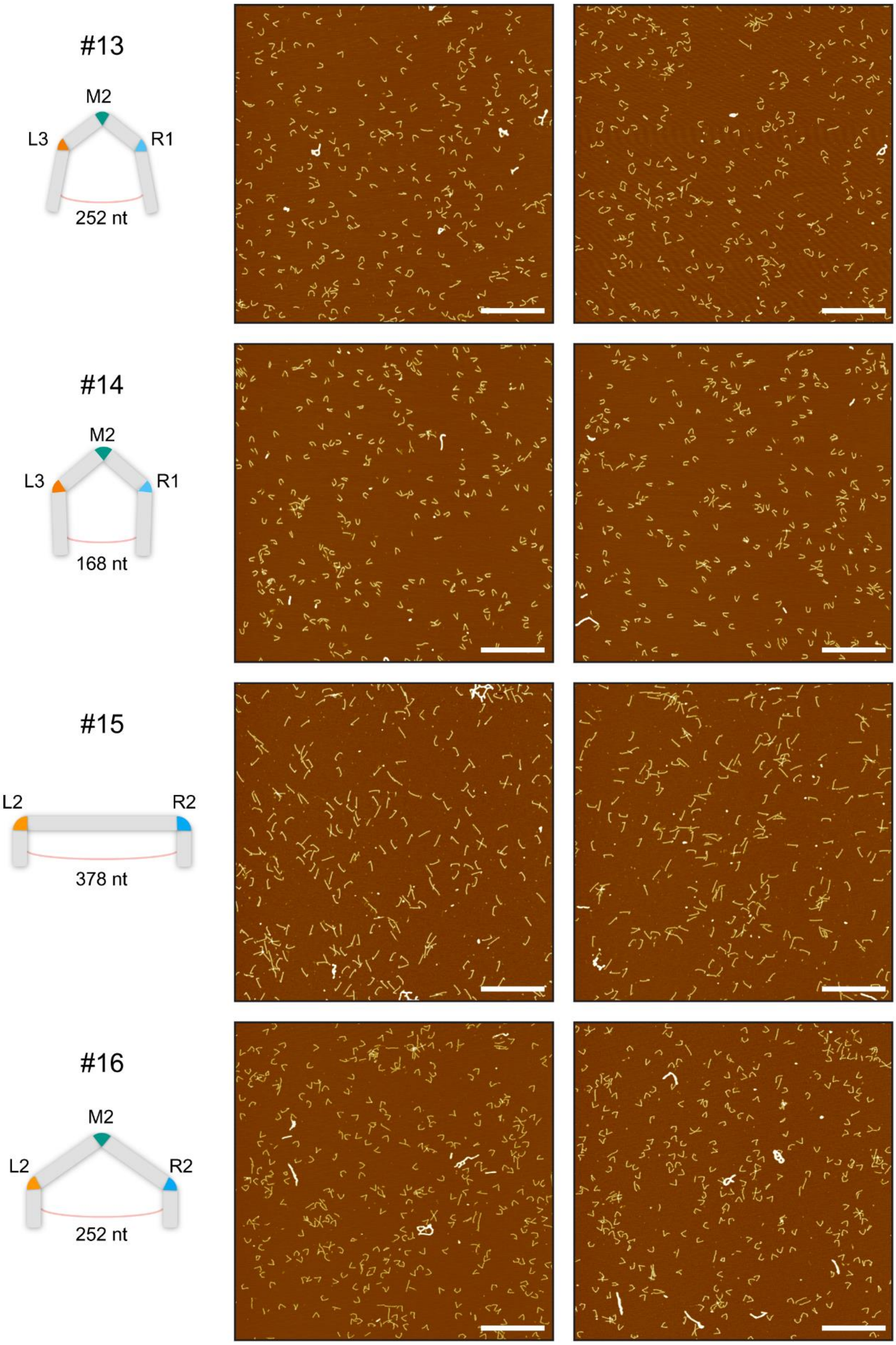

Supplementary Figure 11. AFM images of the structures #13 to #16 shown in Fig. 2. Scale bars: 1 μm.

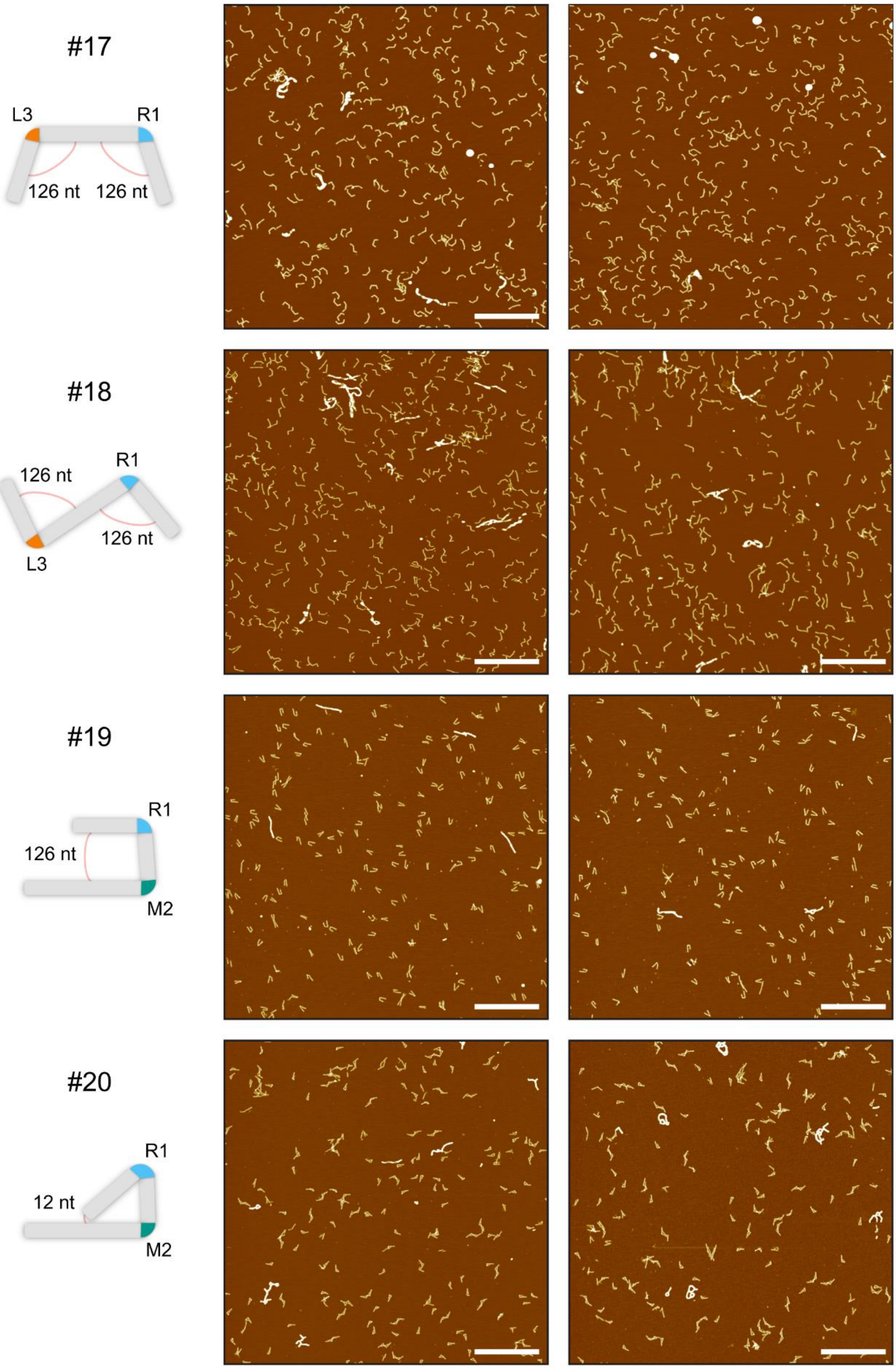

Supplementary Figure 12. AFM images of the structures #17 to #20 shown in Fig. 2. Scale bars: 1 μm.

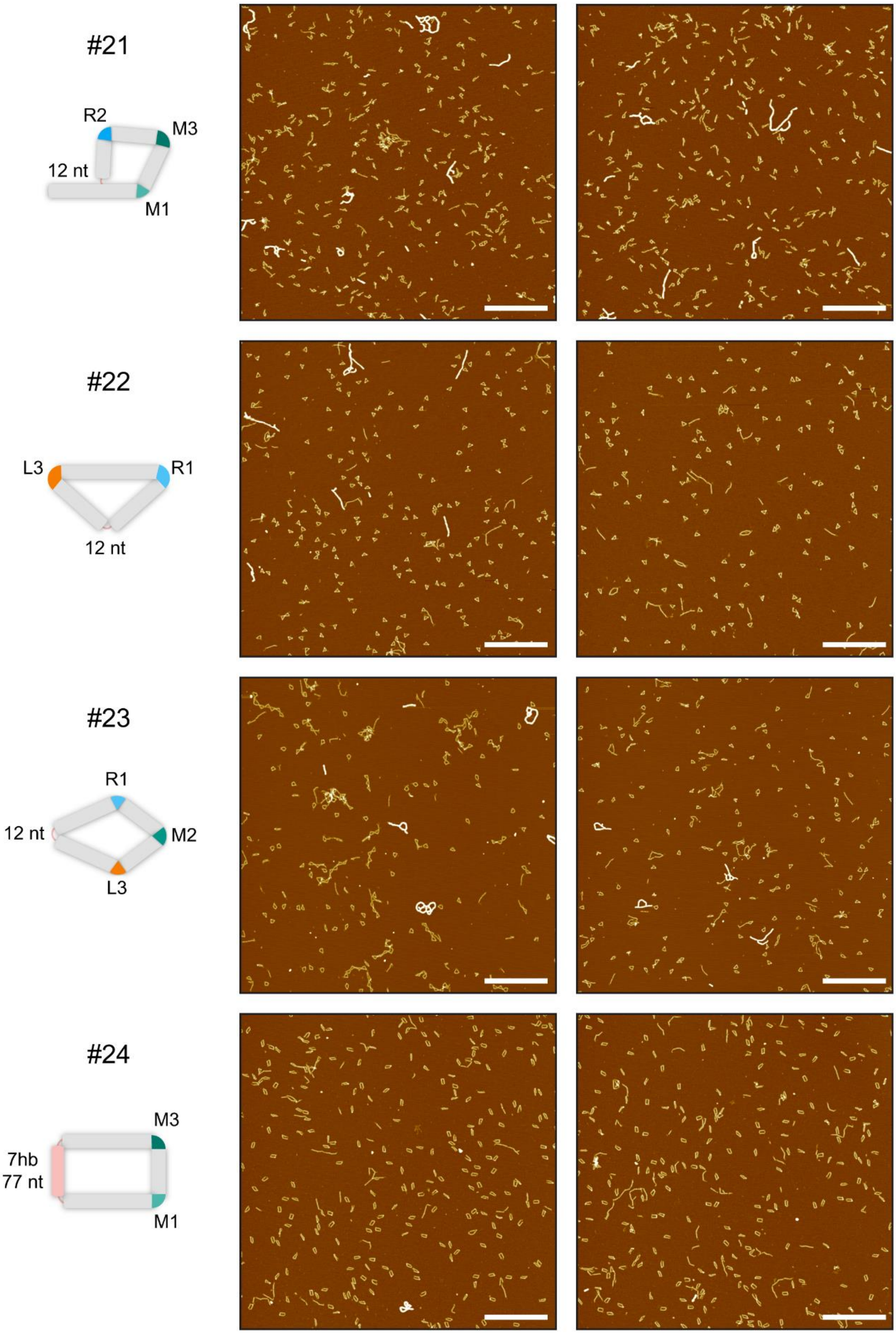

Supplementary Figure 13. AFM images of the structures #21 to #24 shown in Fig. 2. Scale bars: 1 μm.

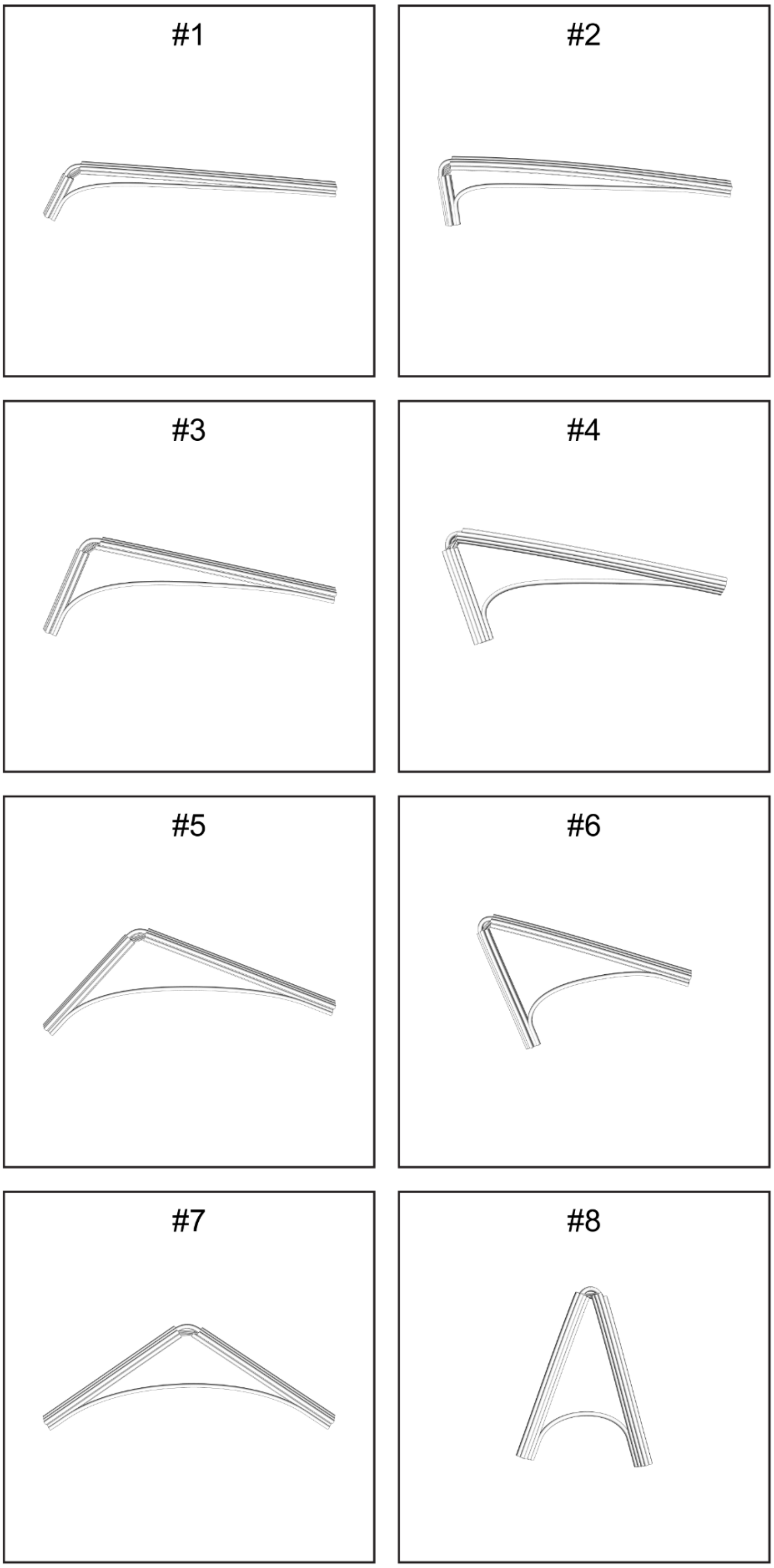

Supplementary Figure 14. CanDo shape prediction of the structures #1 to #8 in Fig. 2.

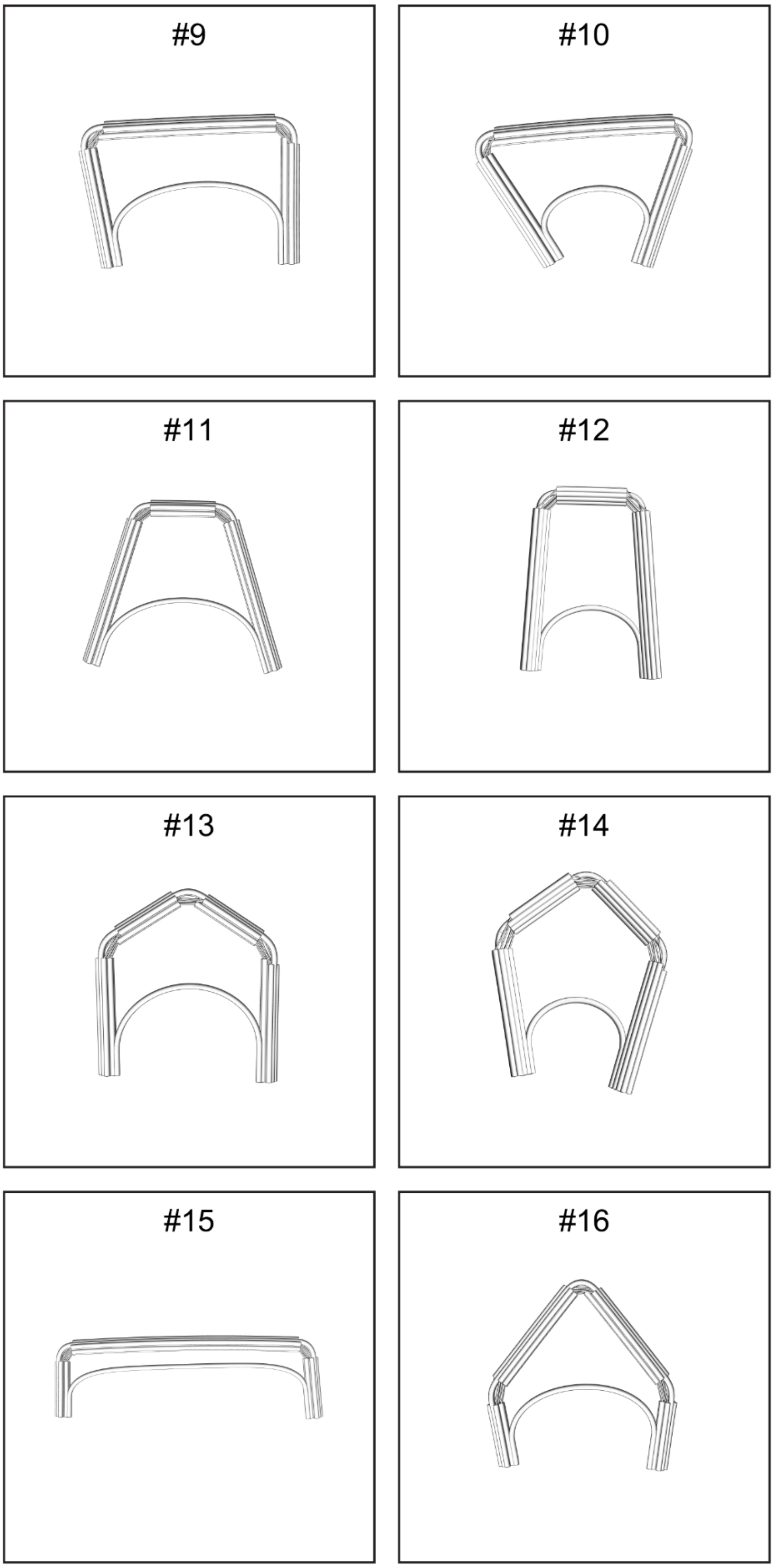

Supplementary Figure 15. CanDo shape prediction of the structures #9 to #16 in Fig. 2.

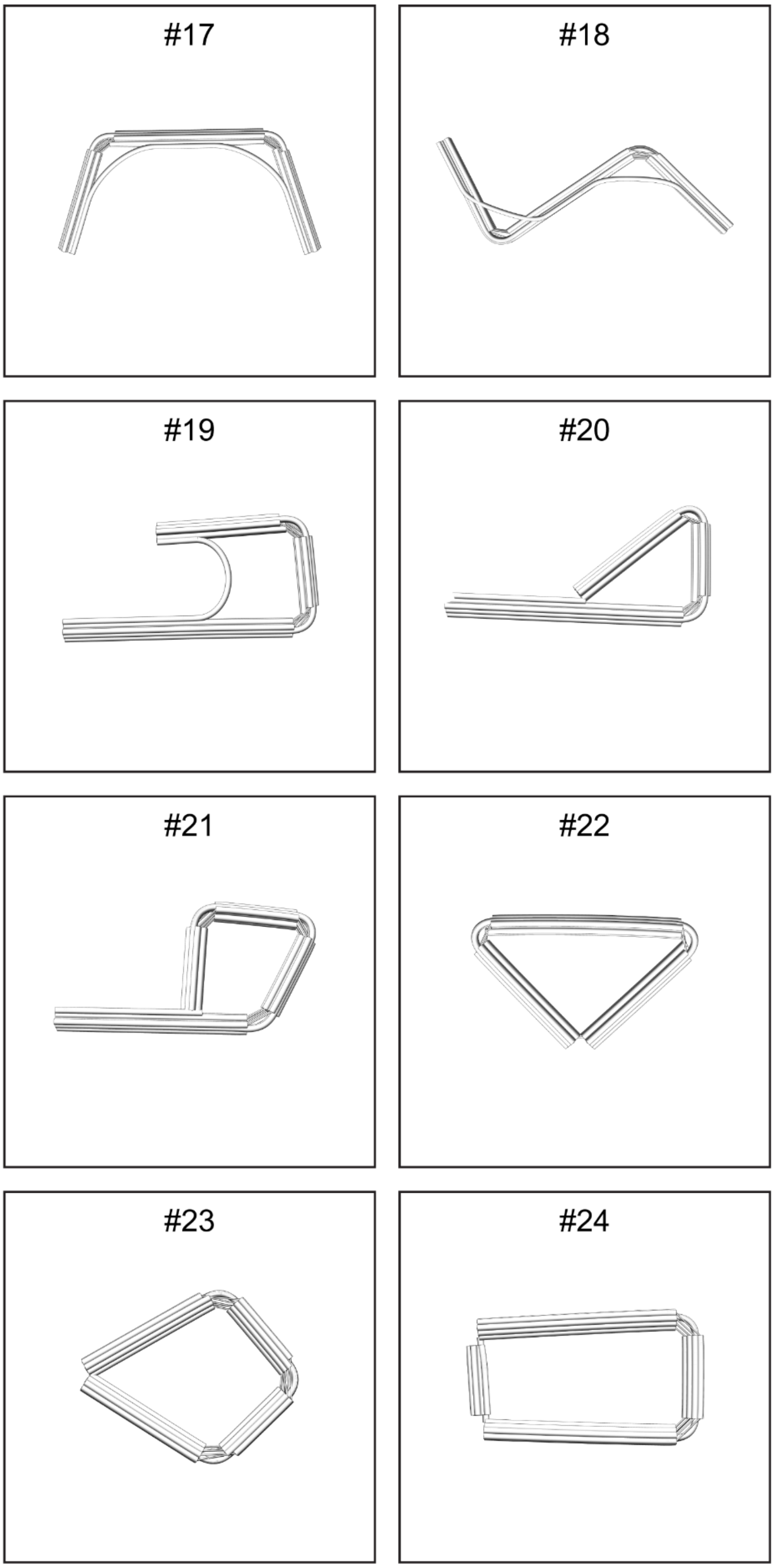

Supplementary Figure 16. CanDo shape prediction of the structures #17 to #24 in Fig. 2.

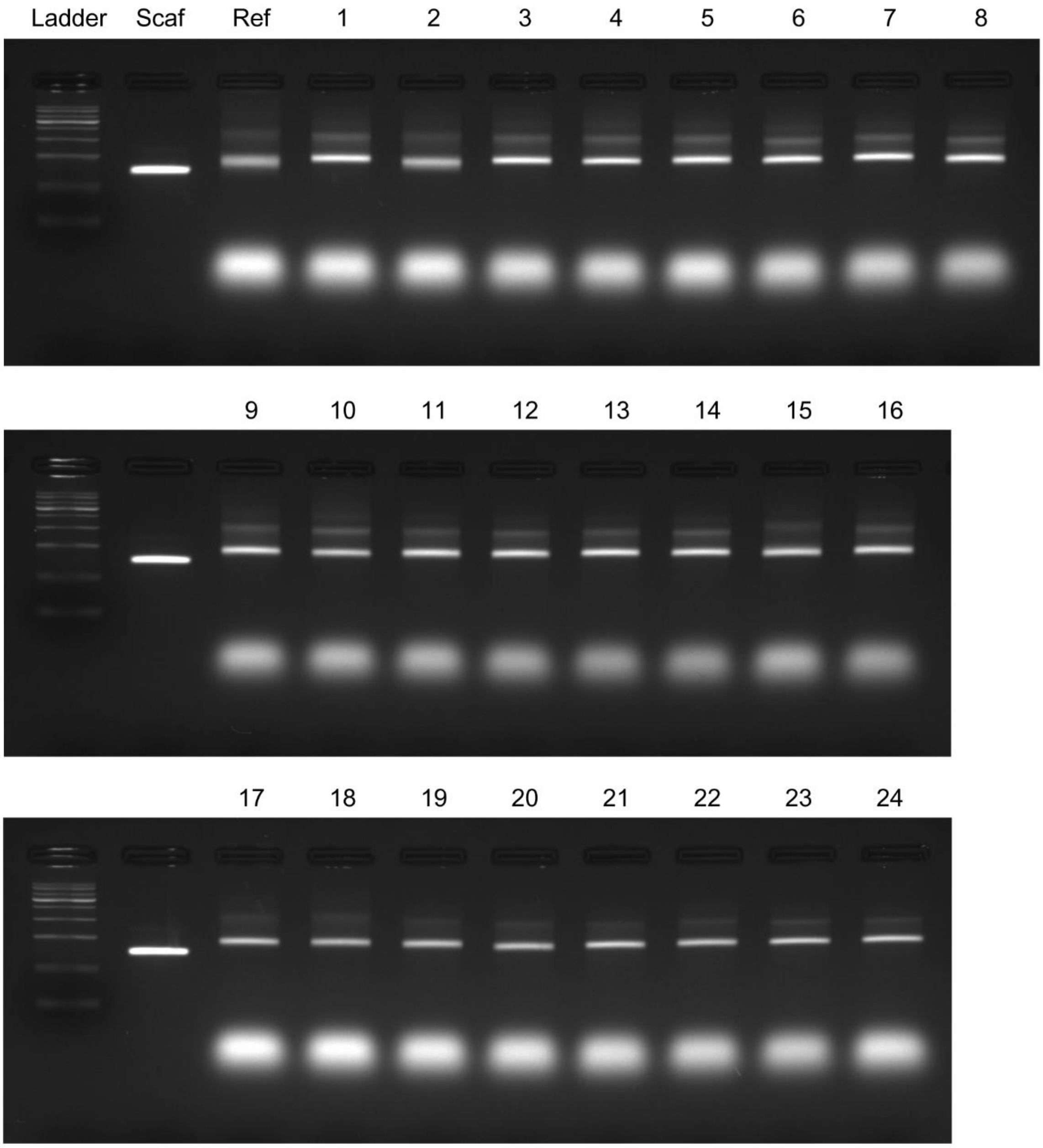

Supplementary Figure 17. Agarose gel electrophoresis results showing the folding yield of the structures shown in Fig. 2.

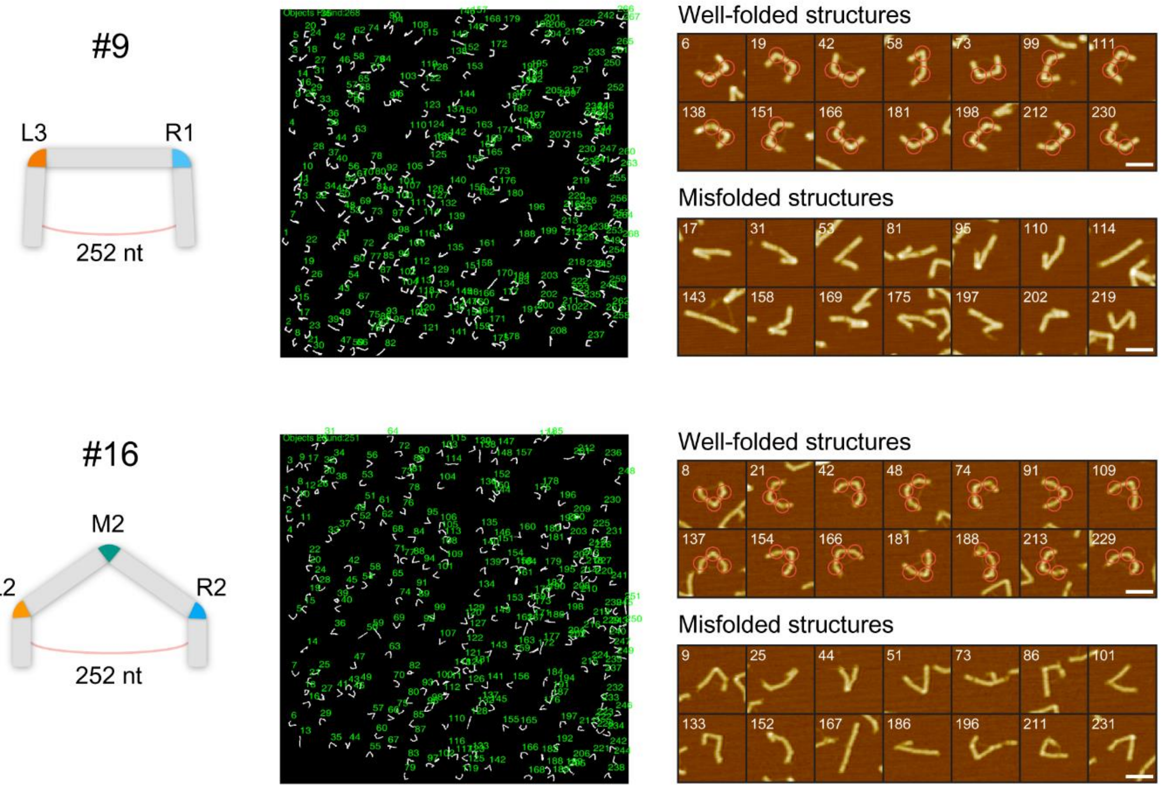

Supplementary Figure 18. Structural folding yield analysis using AFM images. Analysis was performed to all 24 variants in Fig. 2, and two representative double- and triple-hinged structures are shown. After filtering aggregated structures and sediment particles by their sizes, well-folded monomer structures were manually chosen. Well-folded structure should have all hinges bent towards proper direction and amount (shown as orange circles), in accordance with the schematic design. Misfolded structures have either less number of bent region or have hinge(s) bent to opposite direction. Scale bars: 100 nm.

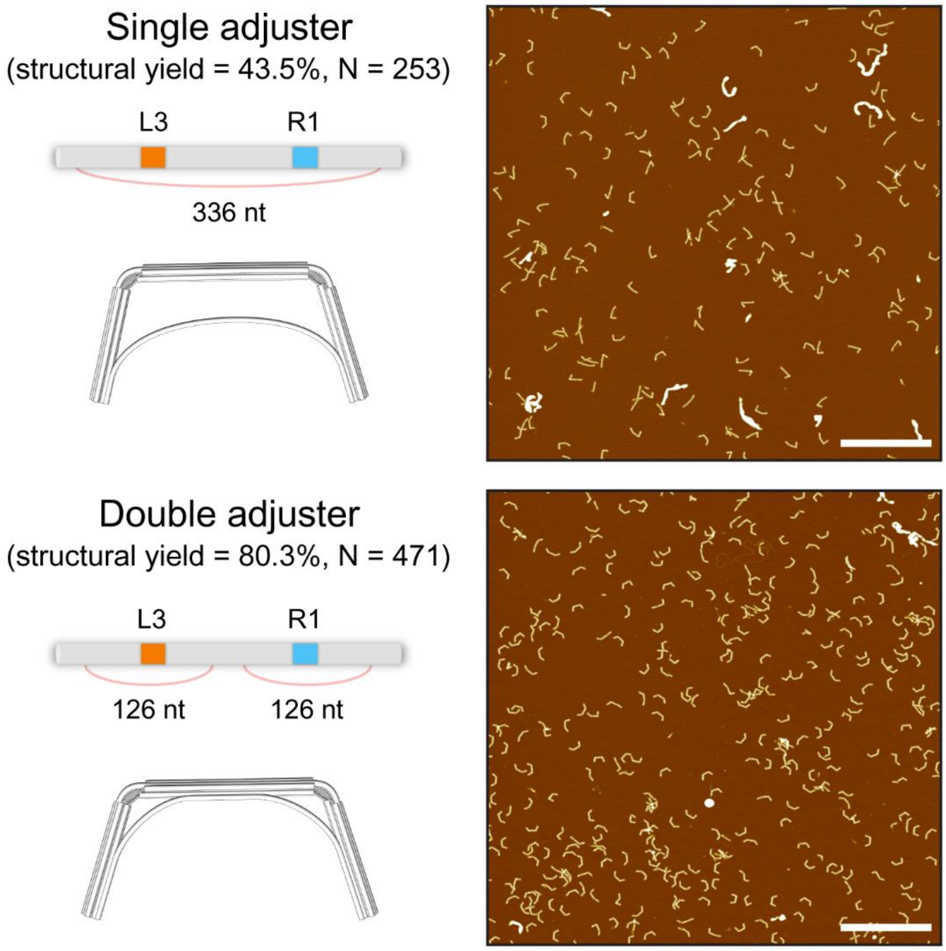

Supplementary Figure 19. An exemplary method to circumvent local energy minima issue in the double-hinged structure with a long adjuster strand. Although CanDo analysis of both designs predicted equally bent shape, single adjuster design showed less portion of correctly folded structures in the experimental result. On the other hand, structural folding yield was significantly enhanced by the separation of the adjuster strand. Scale bars: 1 μm.

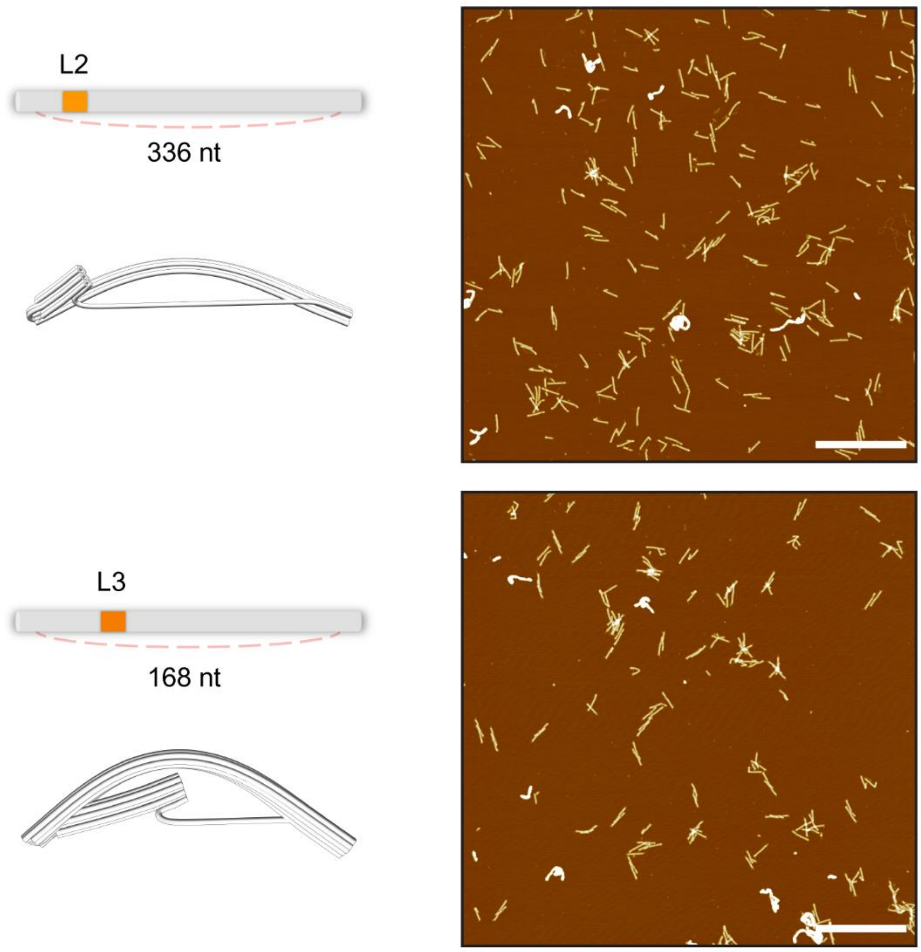

Supplementary Figure 20. Examples of structural failure due to the geometrical distortion. CanDo estimation predicted the collapse of the structure, and the experimental result showed low structural folding yield as well. Scale bars: 1 μm.

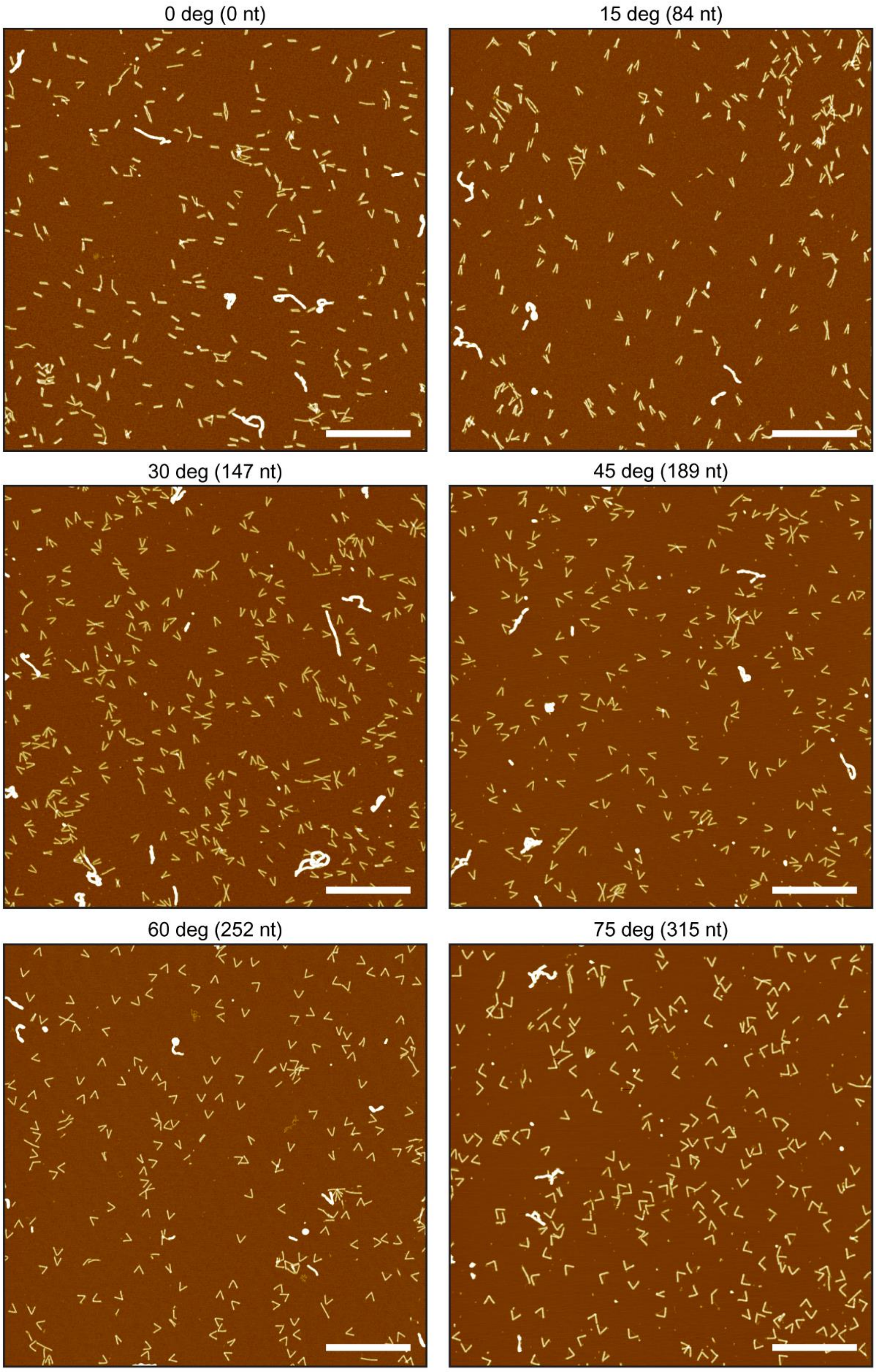

Supplementary Figure 21. Representative AFM images of the structures with the included angle variation from 0° to 75° shown in Fig. 3. Scale bars: 1 μm.

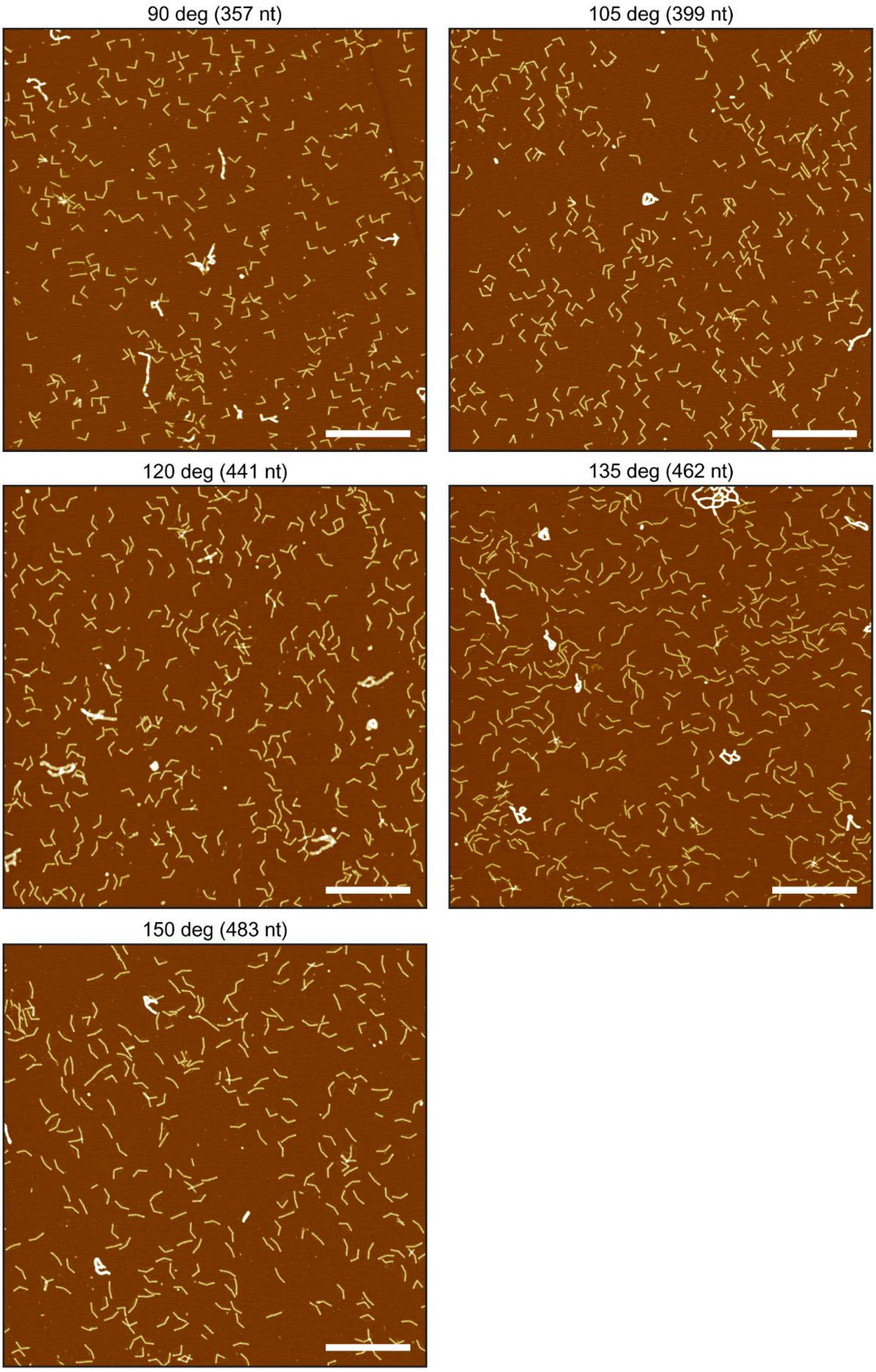

Supplementary Figure 22. Representative AFM images of the structures with the included angle variation from 90° to 150° shown in Fig. 3. Scale bars: 1 μm.

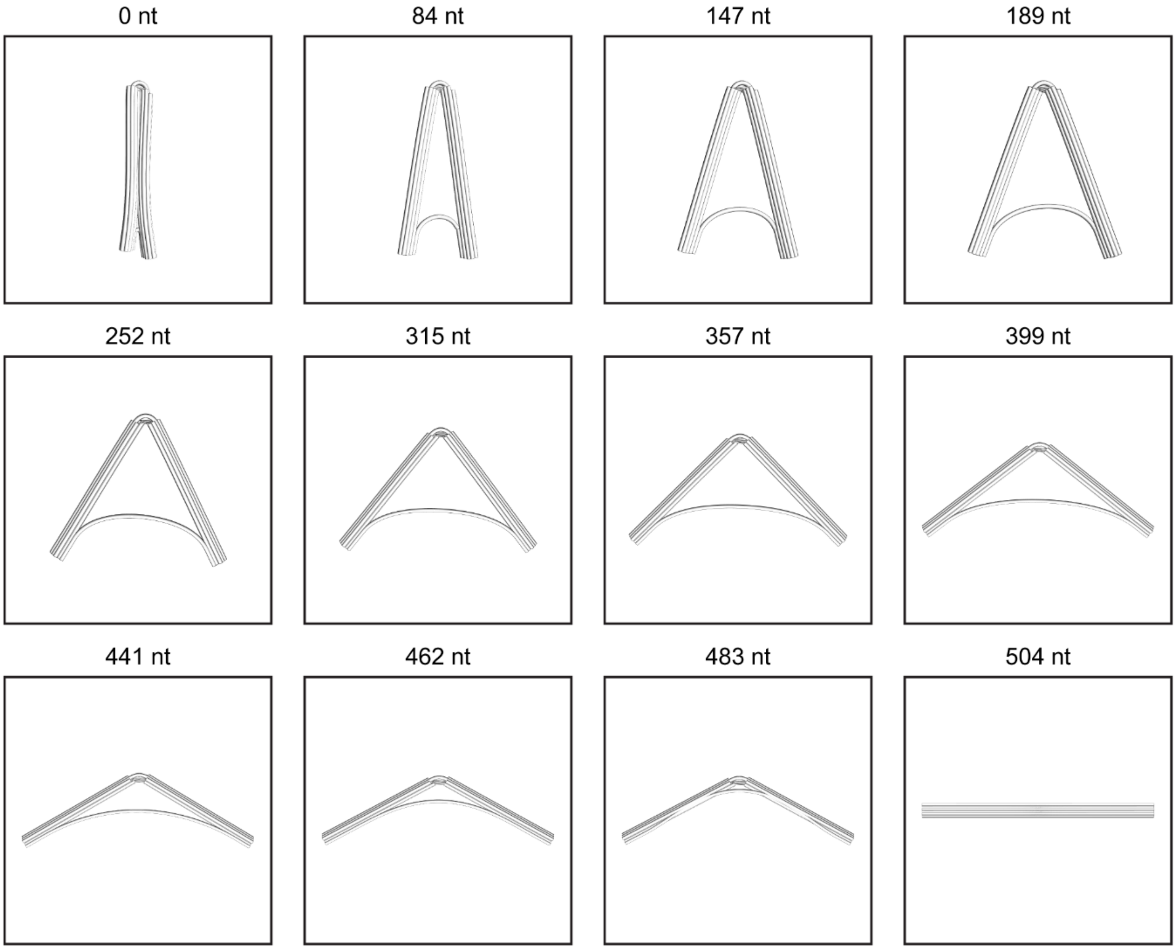

Supplementary Figure 23. CanDo shape prediction of the structures shown in Fig. 3d. Short ssDNAs at the hinge module were included in the analysis.

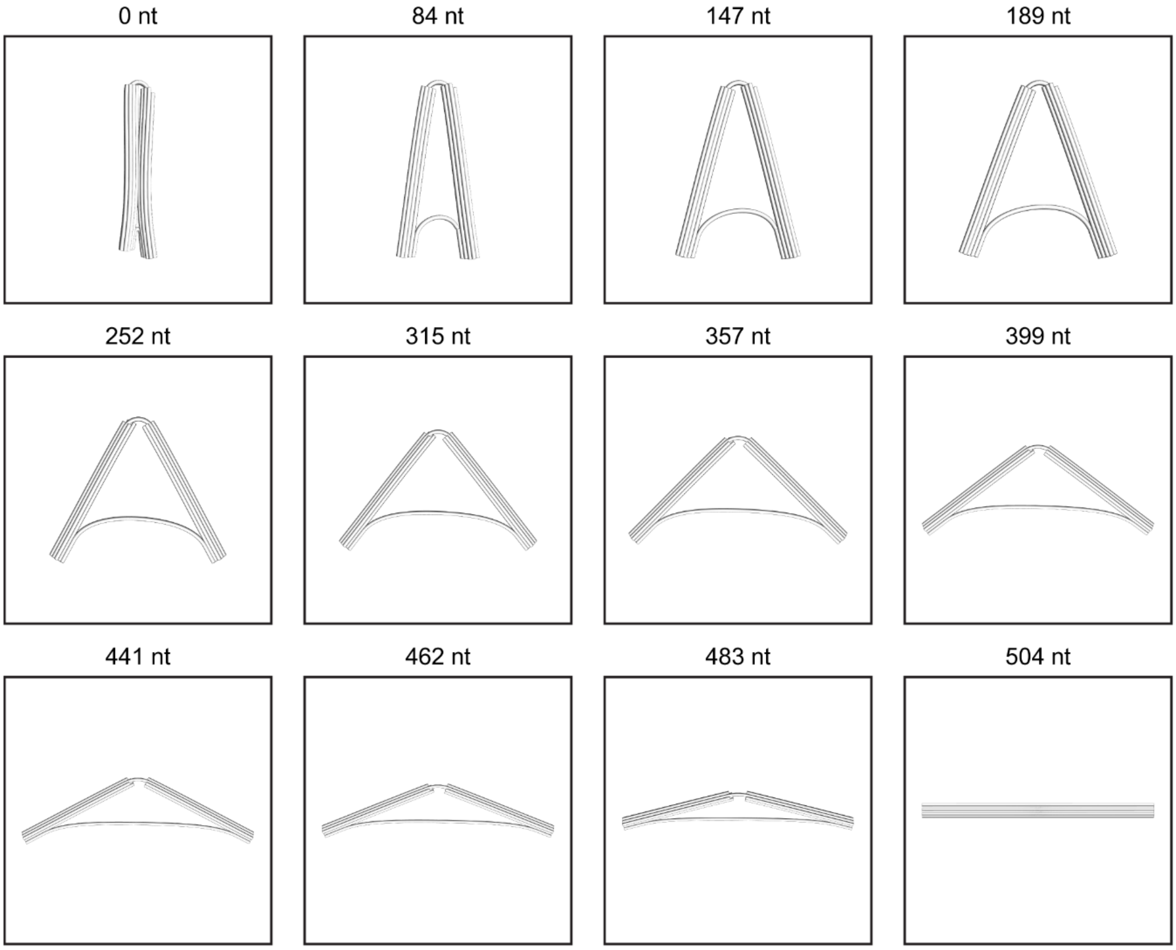

Supplementary Figure 24. CanDo shape prediction of the structures shown in Fig. 3d (w/o hinge ssDNA case). Here, short ssDNAs at the hinge module were excluded in the analysis by removing the ssDNA elements therein.

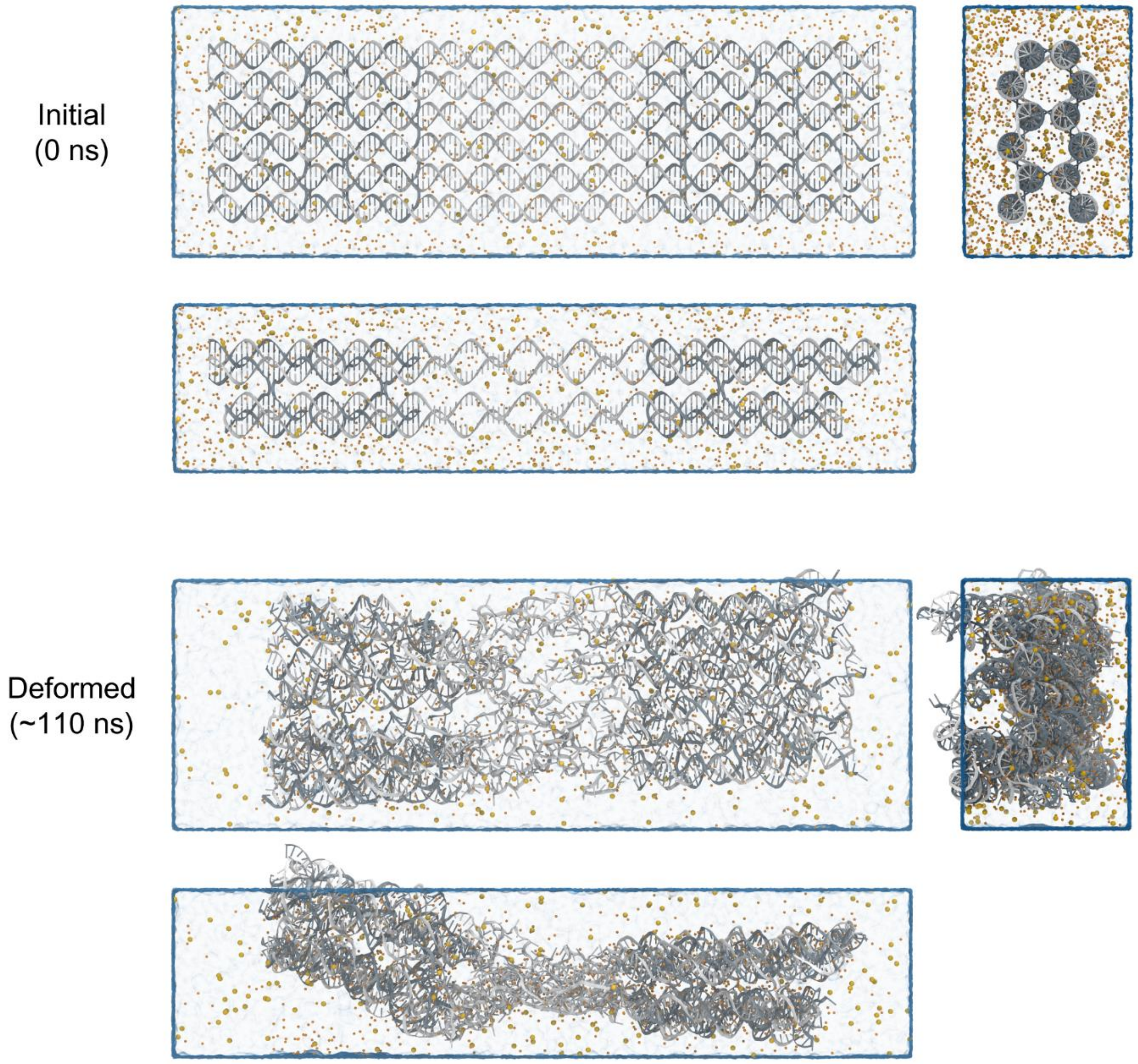

Supplementary Figure 25. MD simulation snapshots showing the initial and deformed (after ~110 ns) configurations of the ds0hb hinge structure.

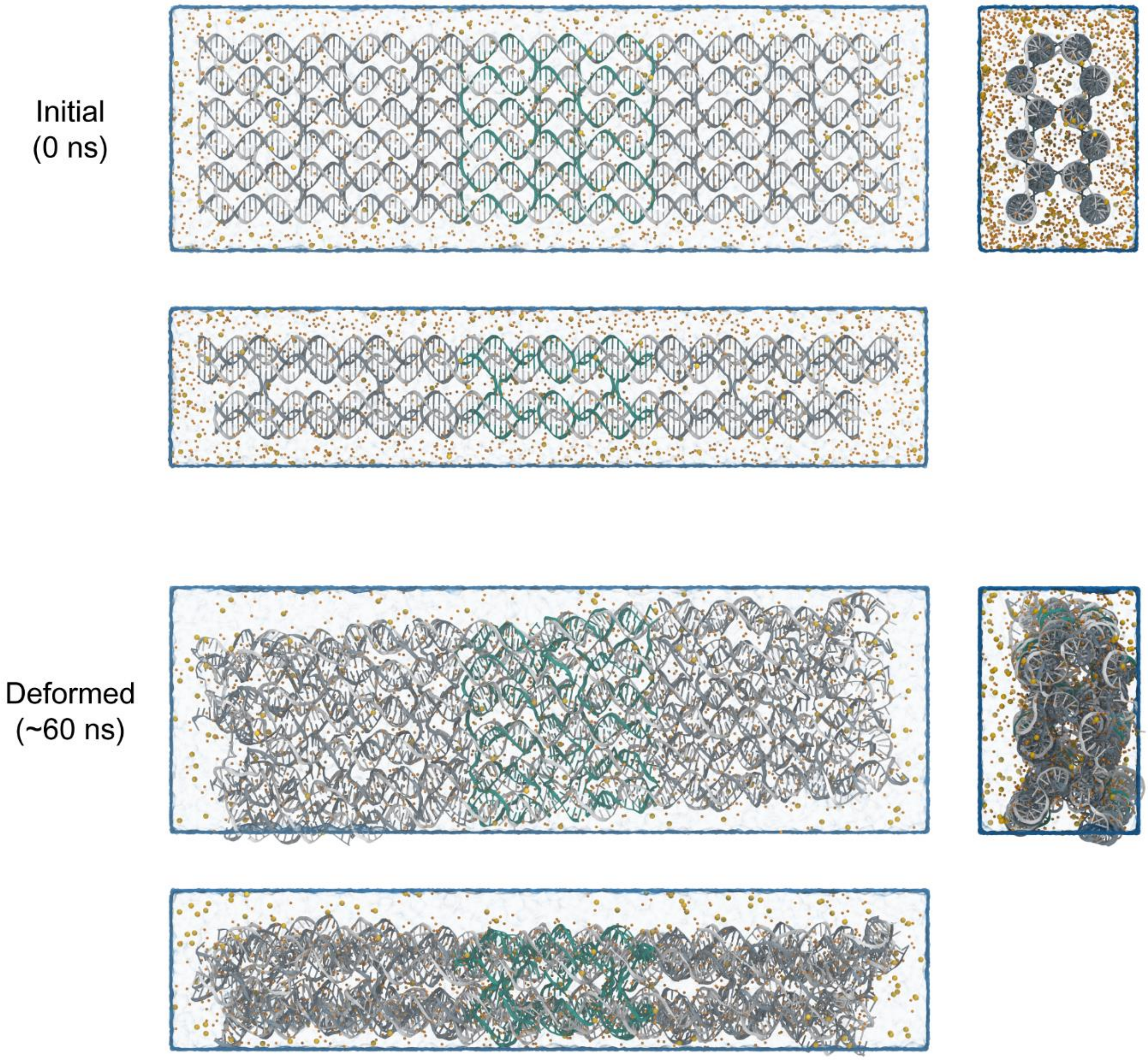

Supplementary Figure 26. MD simulation snapshots showing the initial and deformed (after 60 ns) configurations of the no hinge structure.

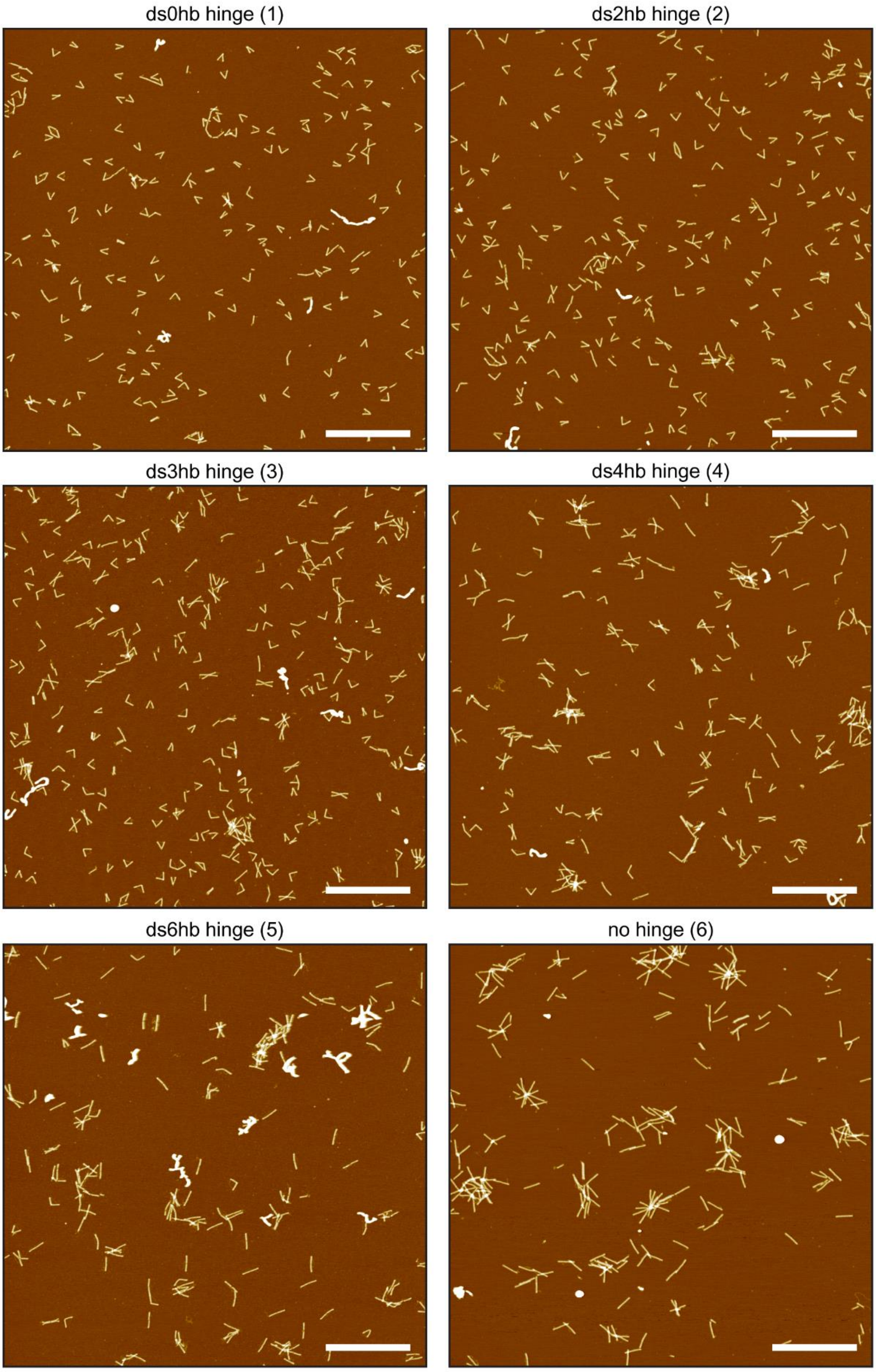

Supplementary Figure 27. Large-area AFM images showing the relationship between hinge stiffness and monomer folding yield in Fig. 5c. Scale bars: 1 μm.

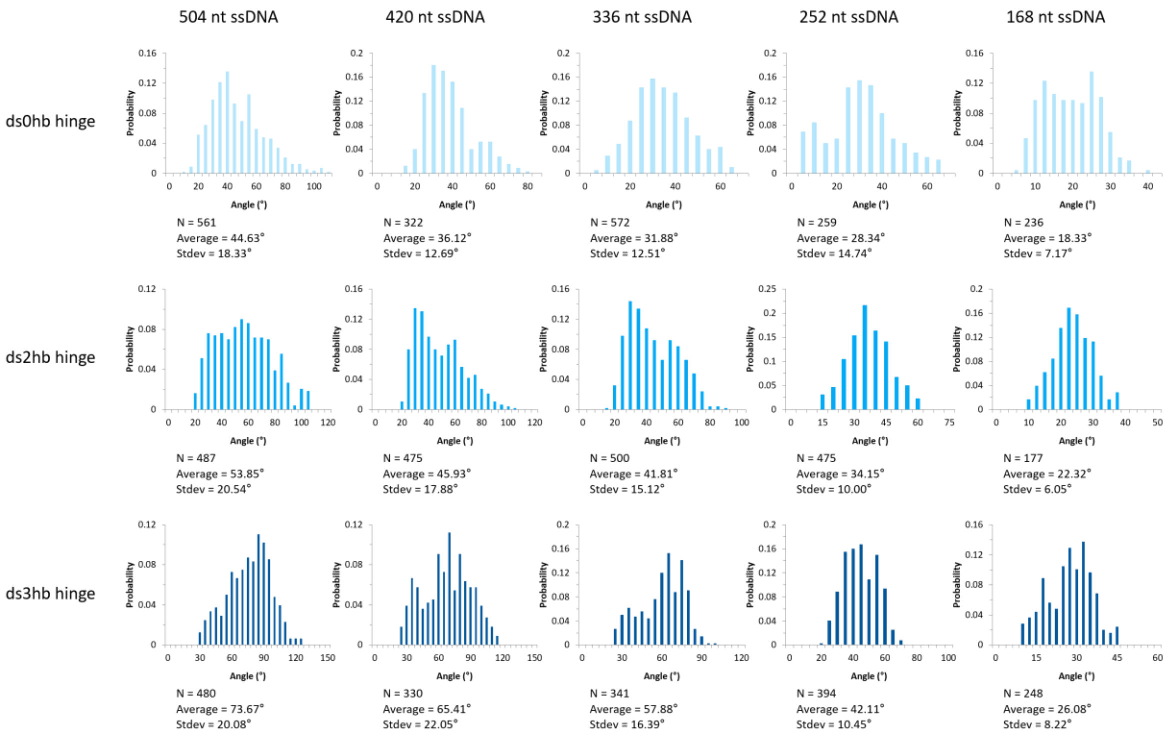

Supplementary Figure 28. Included angle distribution of ssDNA adjuster structures having different adjuster strand length and hinge stiffness.

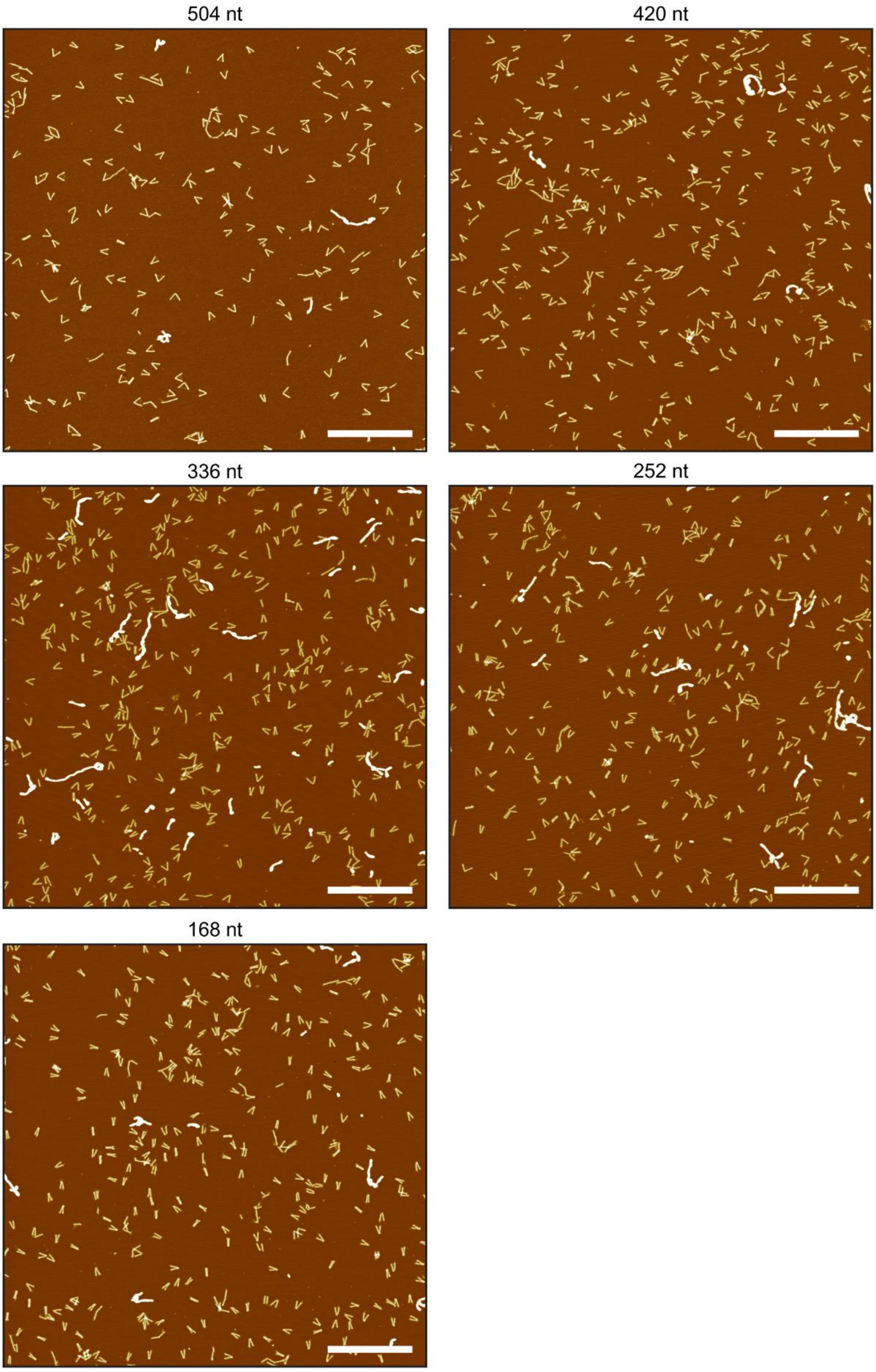

Supplementary Figure 29. AFM images of ds0hb hinge structures with a ssDNA adjuster strand. Scale bars: 1 μm.

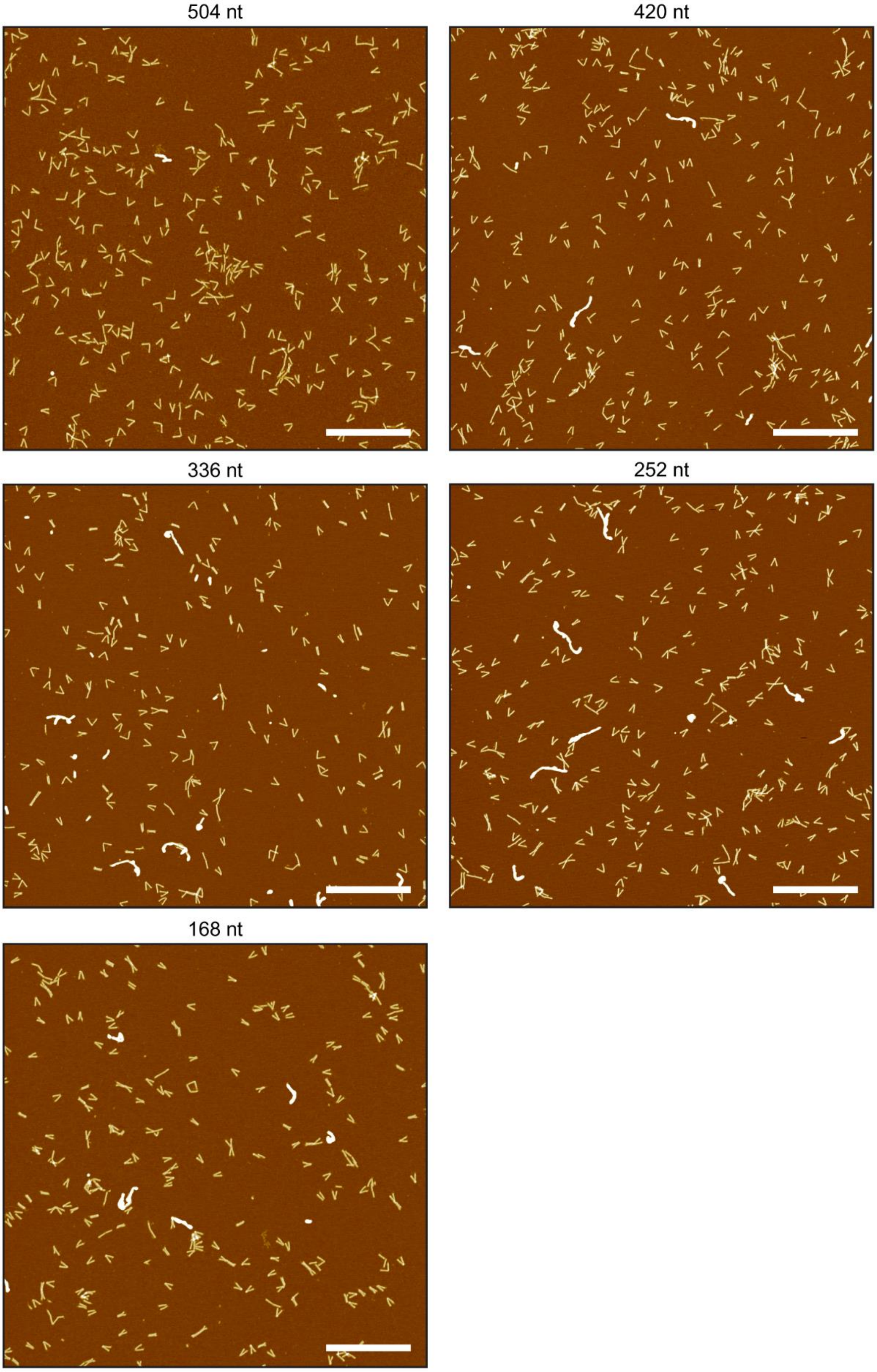

Supplementary Figure 30. AFM images of ds2hb hinge structures with a ssDNA adjuster strand. Scale bars: 1 μm.

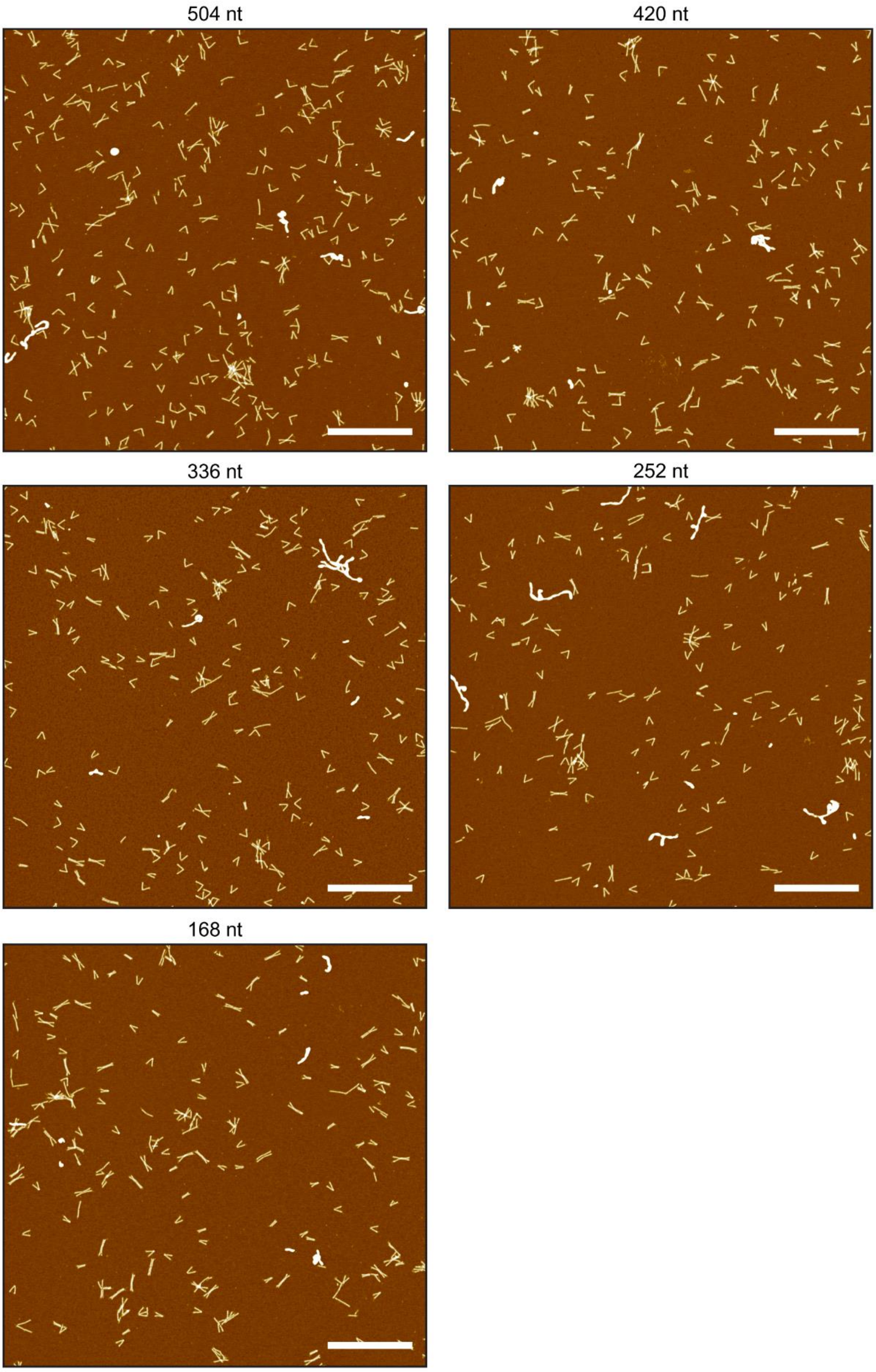

Supplementary Figure 31. AFM images of ds3hb hinge structures with a ssDNA adjuster strand. Scale bars: 1 µm.

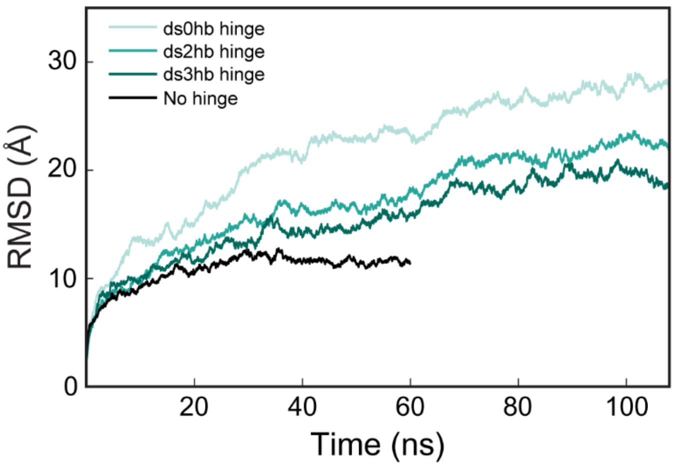

Supplementary Figure 32. RMSD value of all structures during MD simulation.

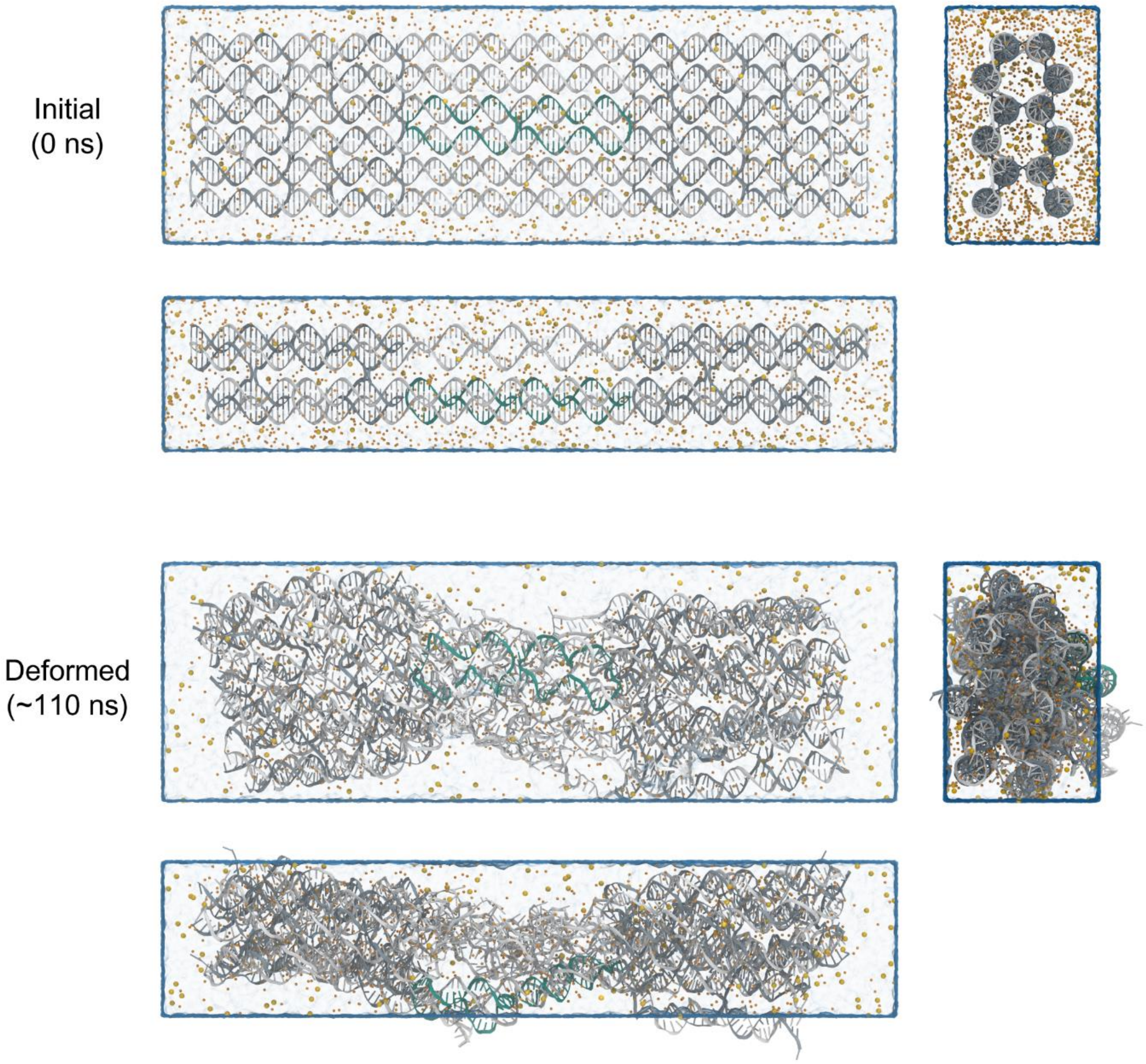

Supplementary Figure 33. MD simulation snapshots showing the initial and deformed (after ~110 ns) configurations of the ds2hb hinge structure.

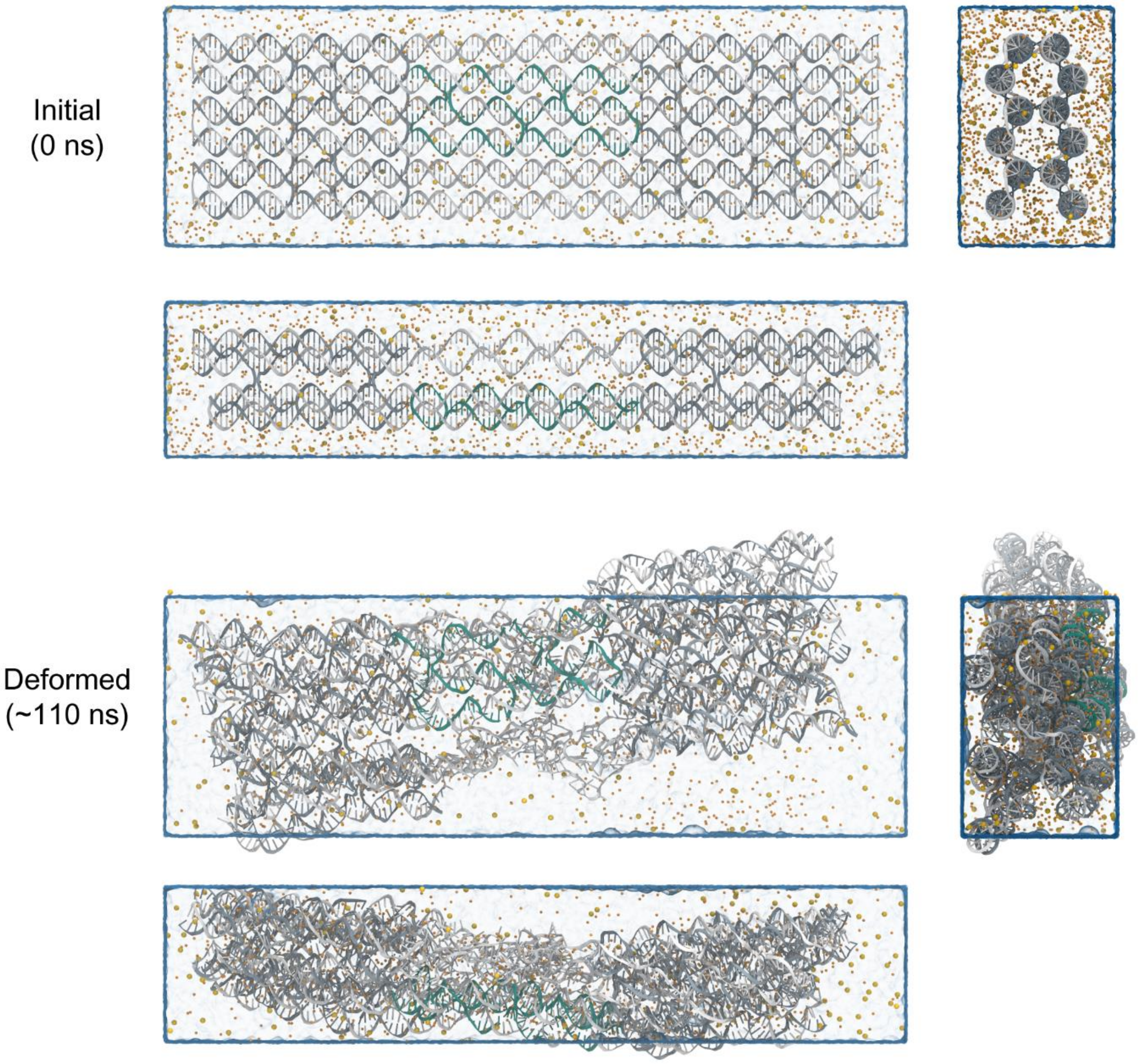

Supplementary Figure 34. MD simulation snapshots showing the initial and deformed (after ~110 ns) configurations of the ds3hb hinge structure.

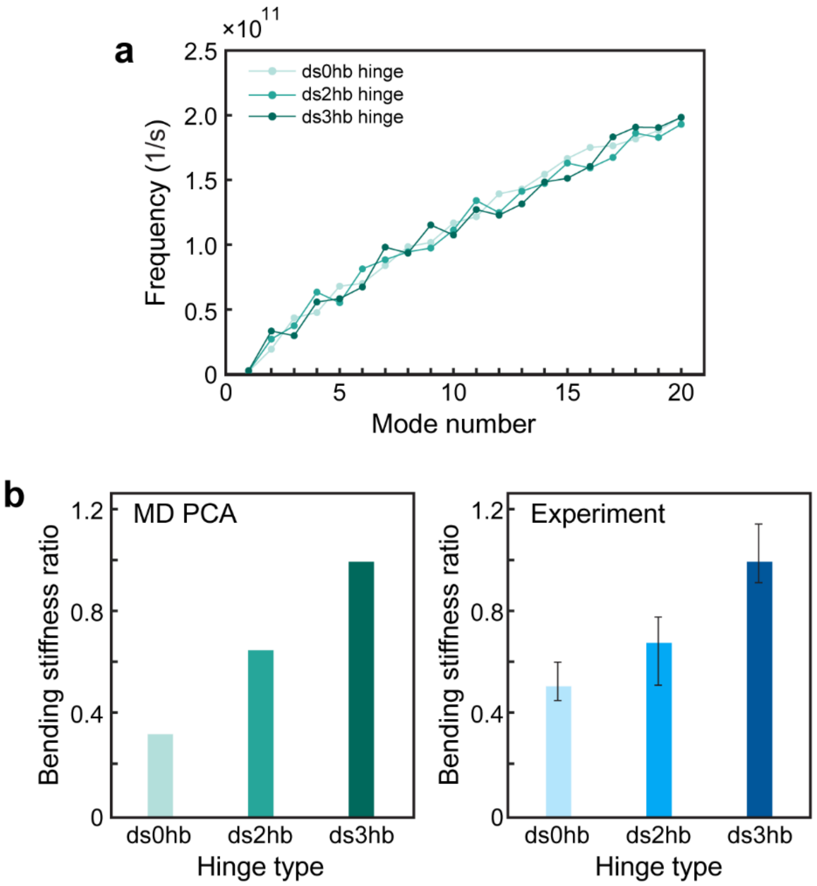

Supplementary Figure 35. Principal component analysis (PCA) from MD simulation. (a) The natural frequencies of all hinged structures. For all cases, the first mode (having smallest natural frequency) was axial stretching and the second mode was first bending mode. (b) Relative bending stiffness ratios calculated from MD trajectories using PCA and measured from experiments shown in Fig. 5e. For each case, bending stiffness of the ds3hb hinge was set to 1 as a reference.

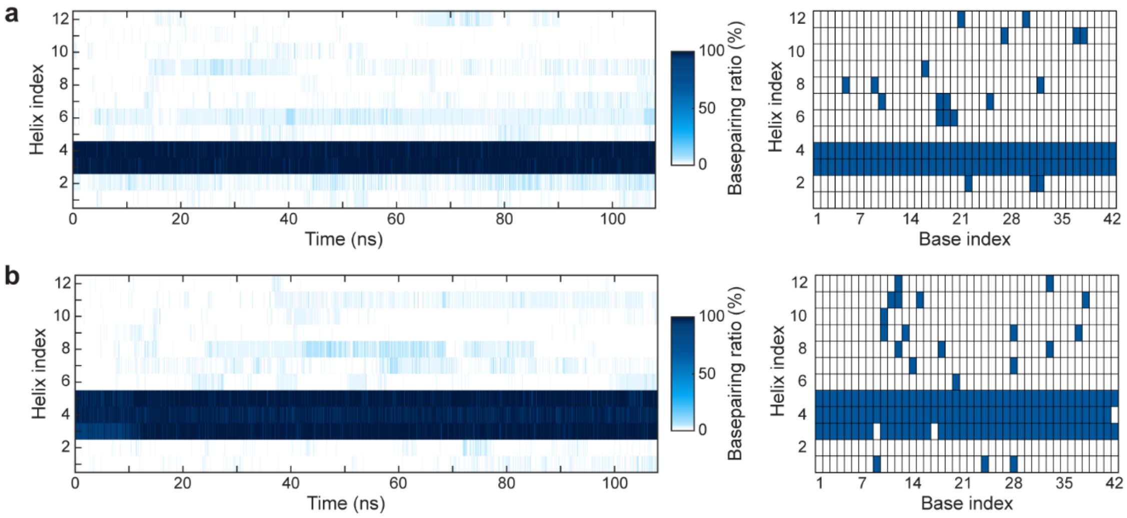

Supplementary Figure 36. Basepairing ratio analysis from MD simulation. Diagrams showing the basepairing ratio of each helix during MD simulation and positions of the individual basepairing at the final time step of ds2hb (a) and ds3hb (b) hinged structures.

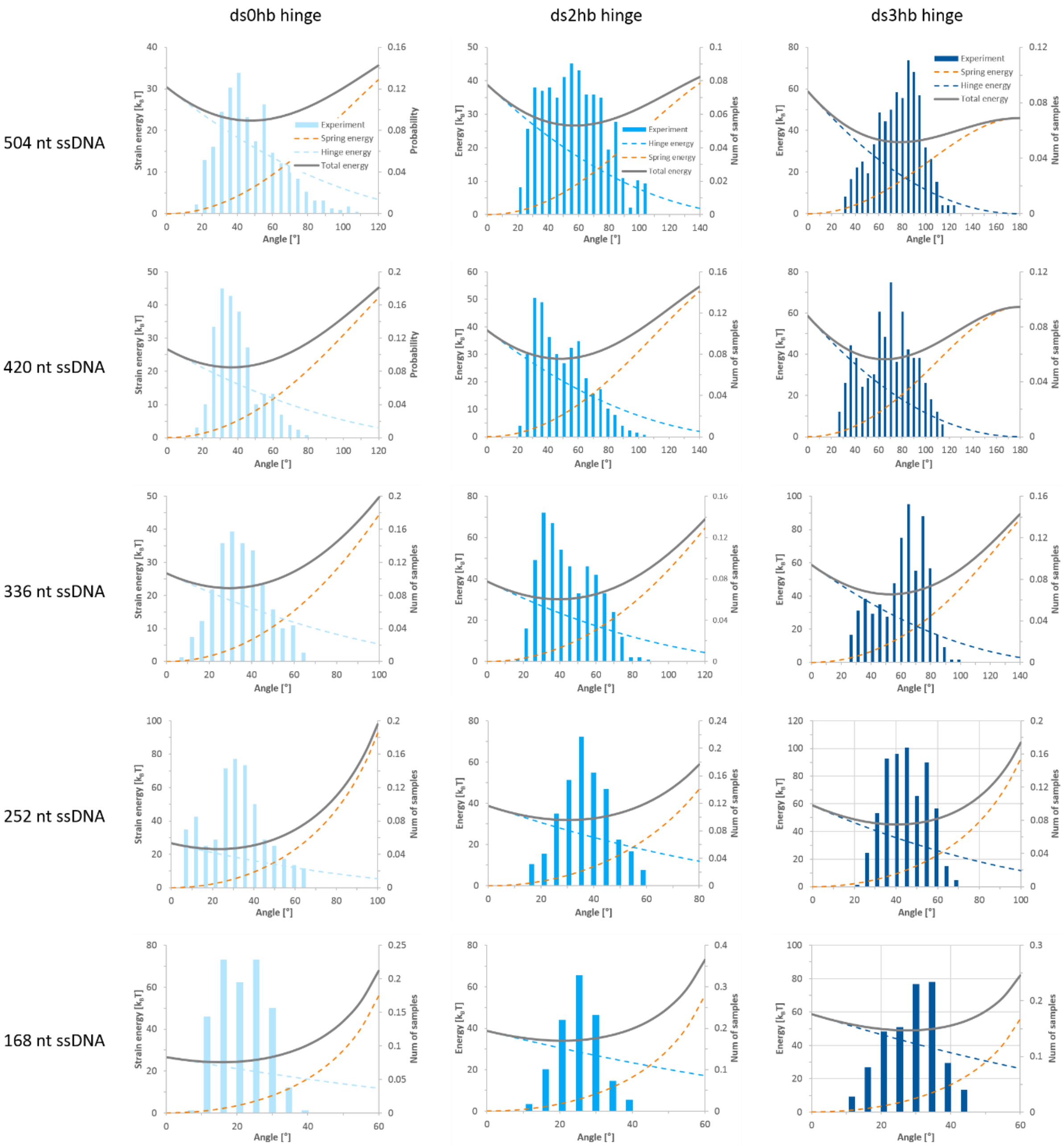

Supplementary Figure 37. Strain energy calculation of the structures having a ssDNA adjuster strand with different length and hinge stiffness.

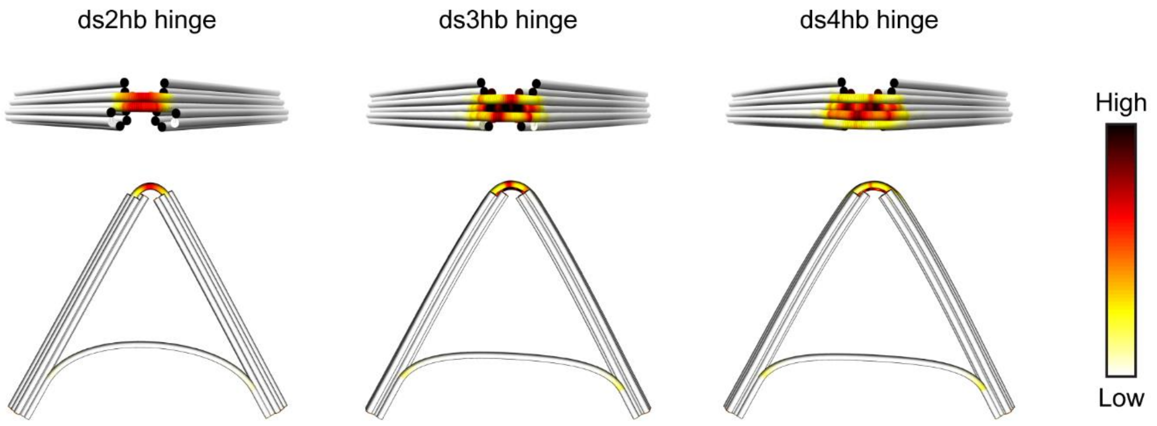

Supplementary Figure 38. Representative CanDo results showing the strain energy concentration at the hinge region. All structures have a 252-nt-long dsDNA adjuster.

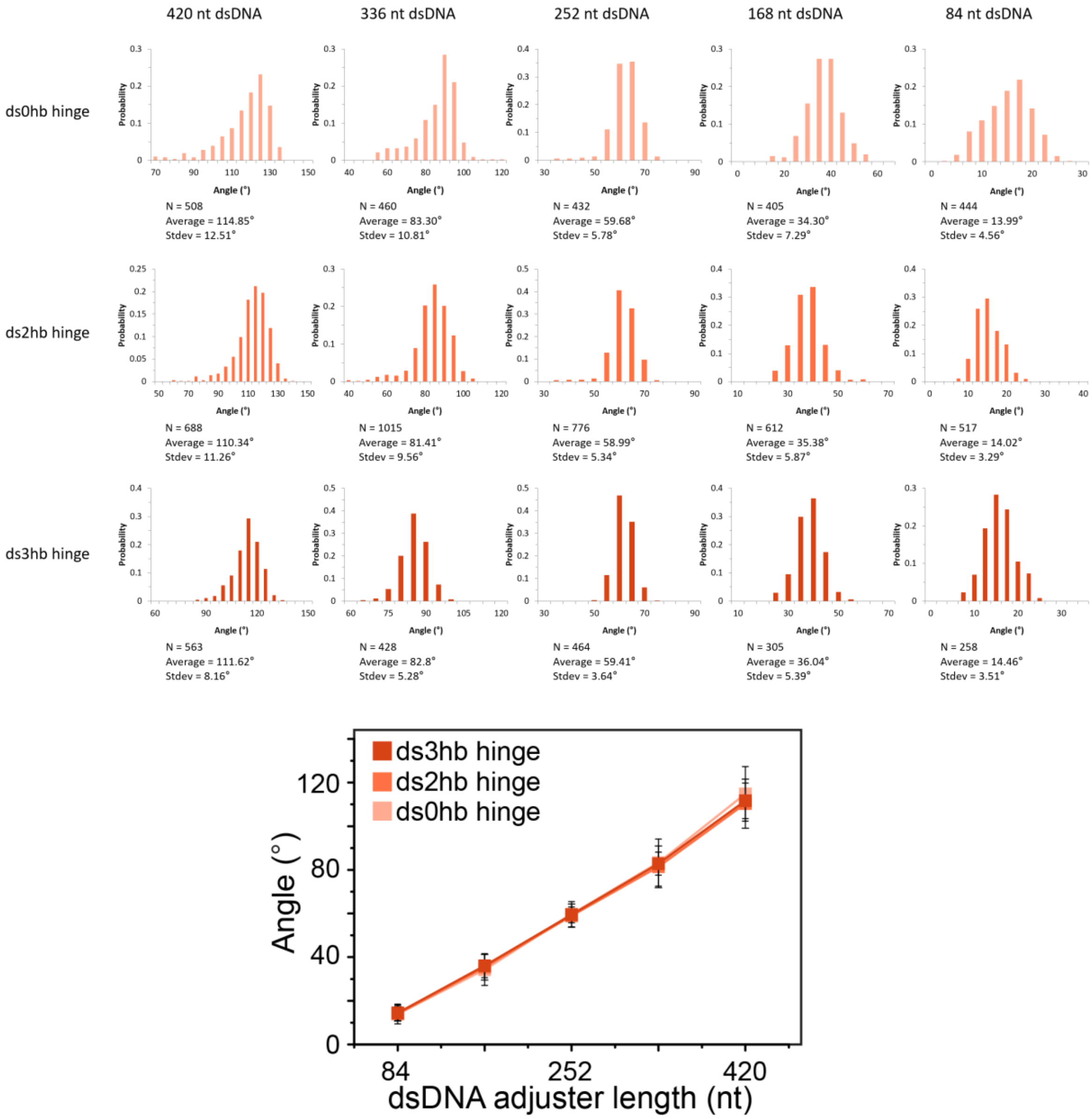

Supplementary Figure 39. Included angle distribution of dsDNA adjuster structures having different adjuster strand length and hinge stiffness. Error bars indicate standard deviation.

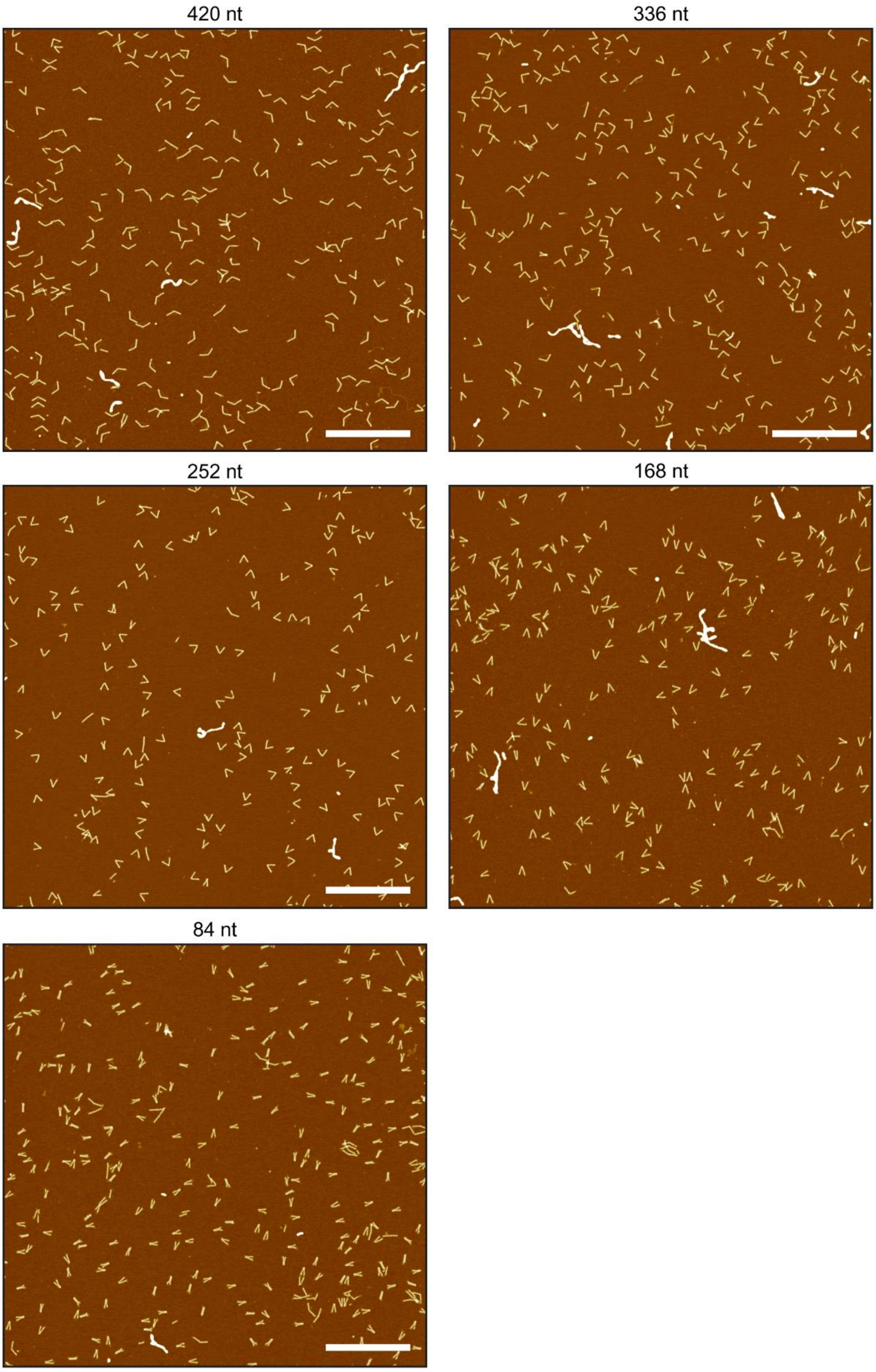

Supplementary Figure 40. AFM images of ds0hb hinge structures with a dsDNA adjuster strand. Scale bars: 1 µm.

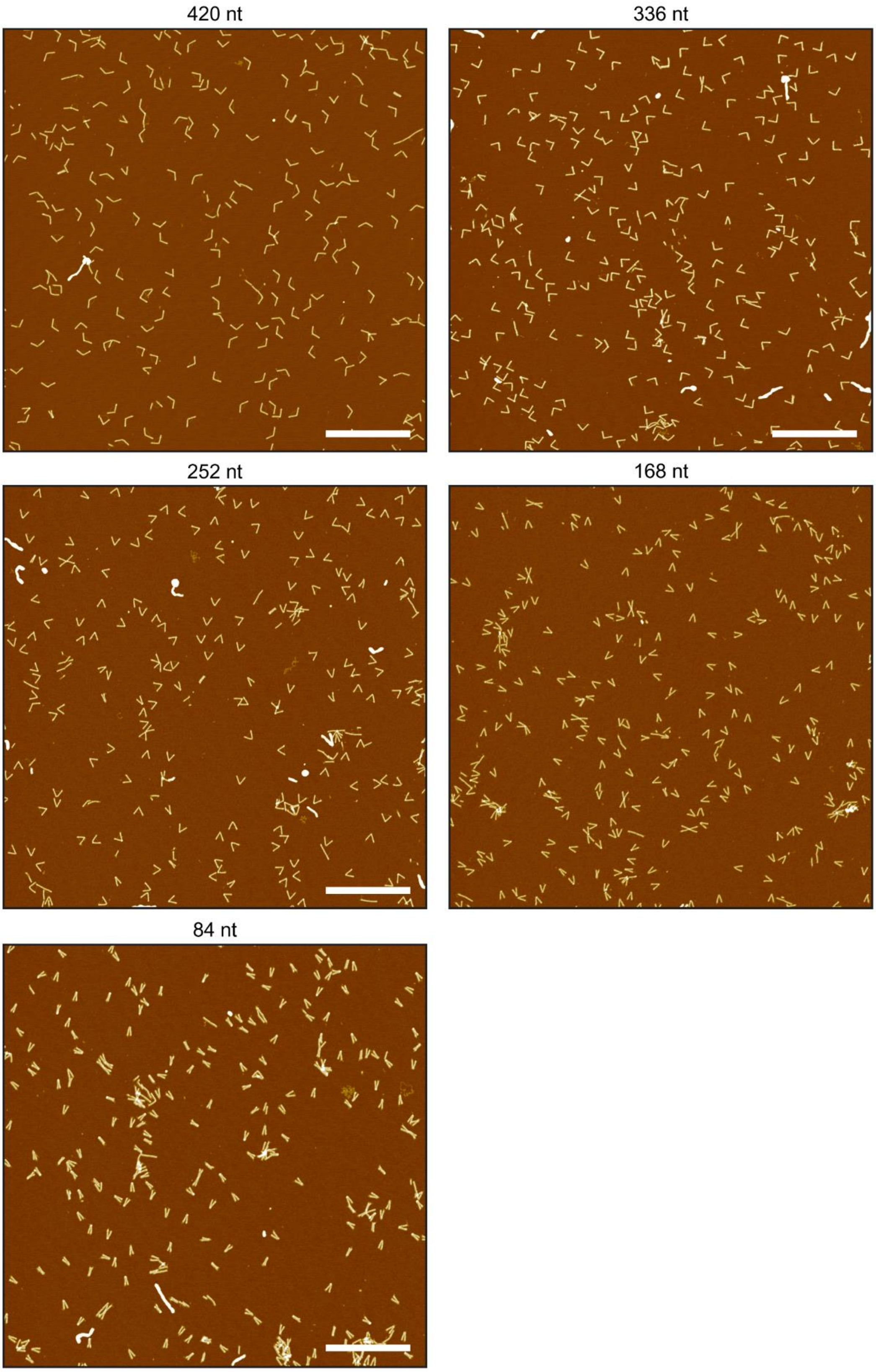

Supplementary Figure 41. AFM images of ds2hb hinge structures with a dsDNA adjuster strand. Scale bars: 1 μm.

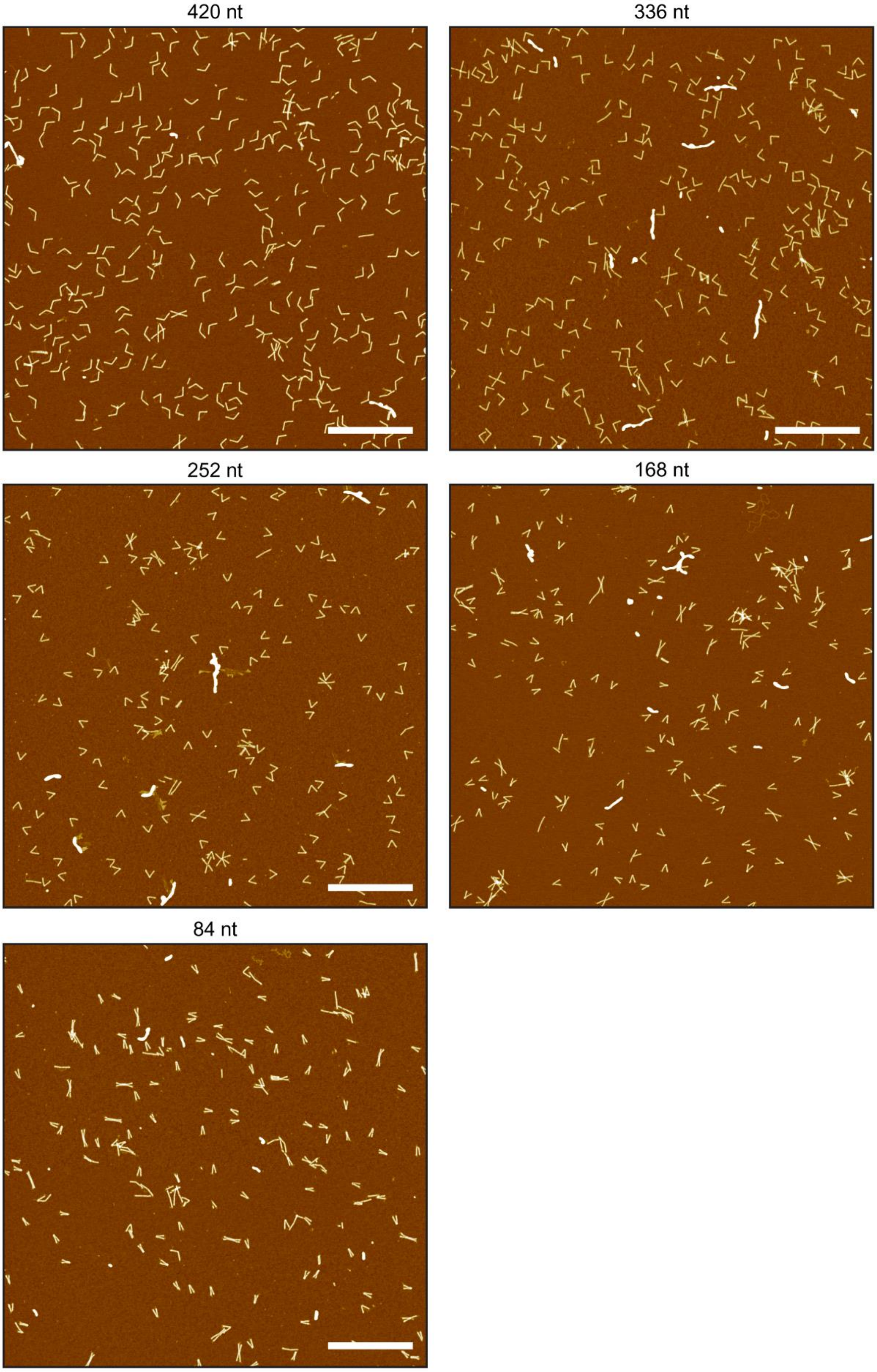

Supplementary Figure 42. AFM images of ds3hb hinge structures with a dsDNA adjuster strand. Scale bars: 1 µm.

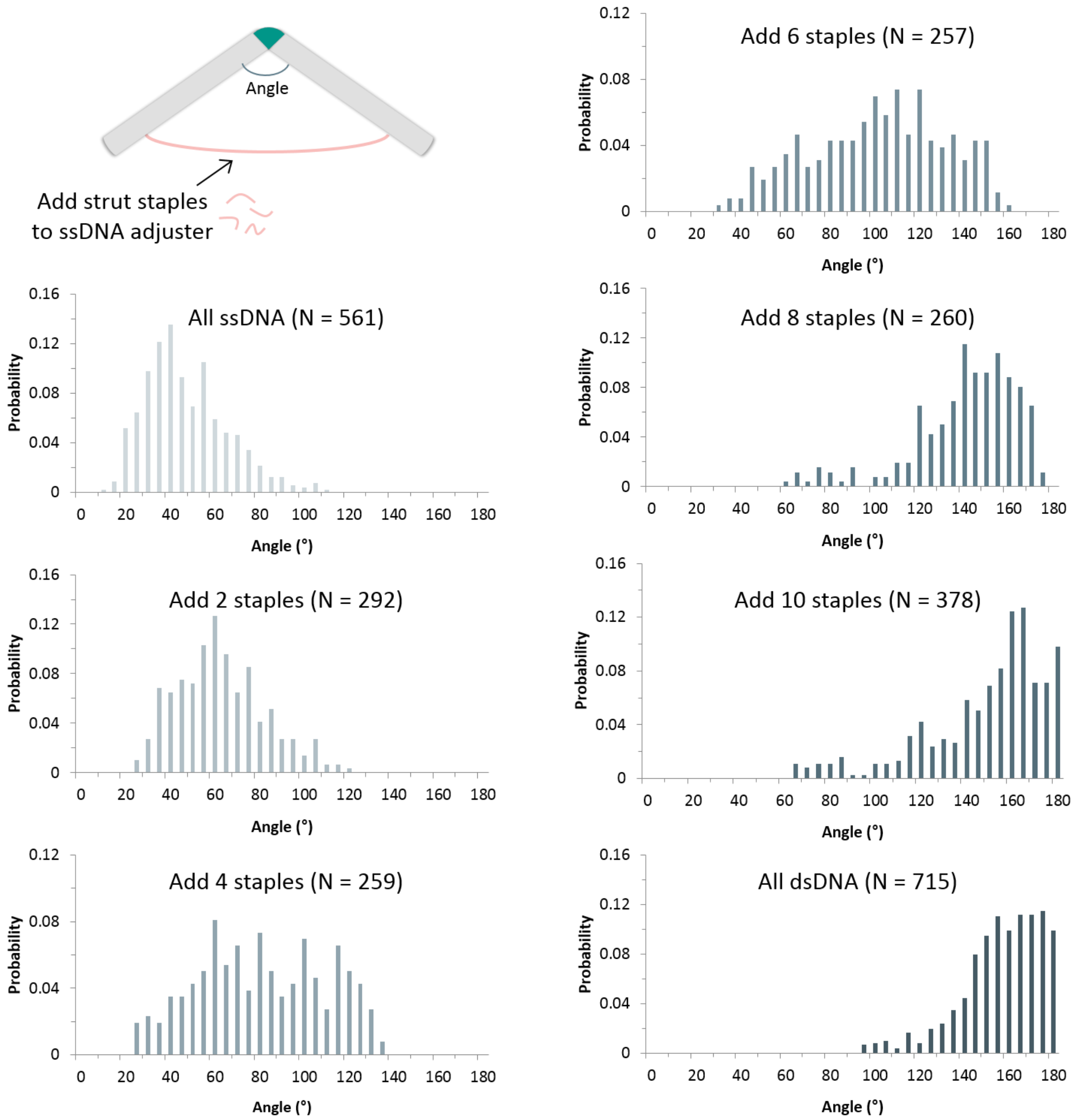

Supplementary Figure 43. Included angle distribution of the ds0hb hinge structures having a different number of adjuster strut staples.

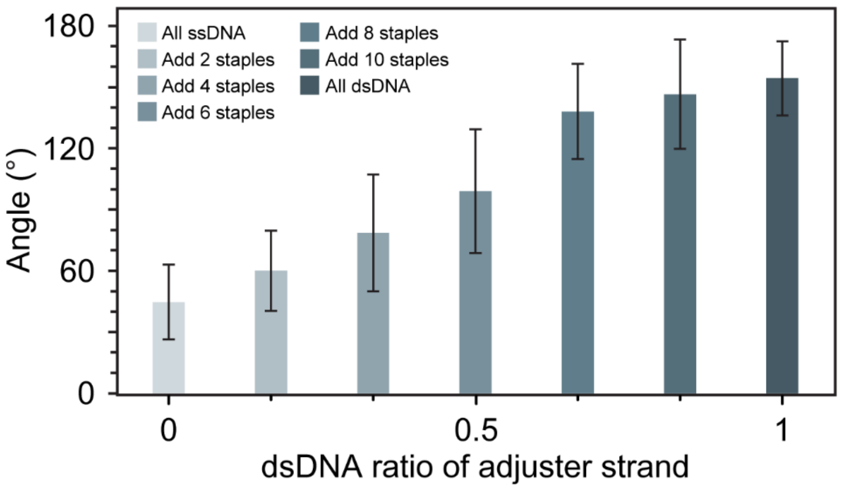

Supplementary Figure 44. Average included angle distribution of the structures having a different number of adjuster strut staples. Error bars indicate standard deviation.

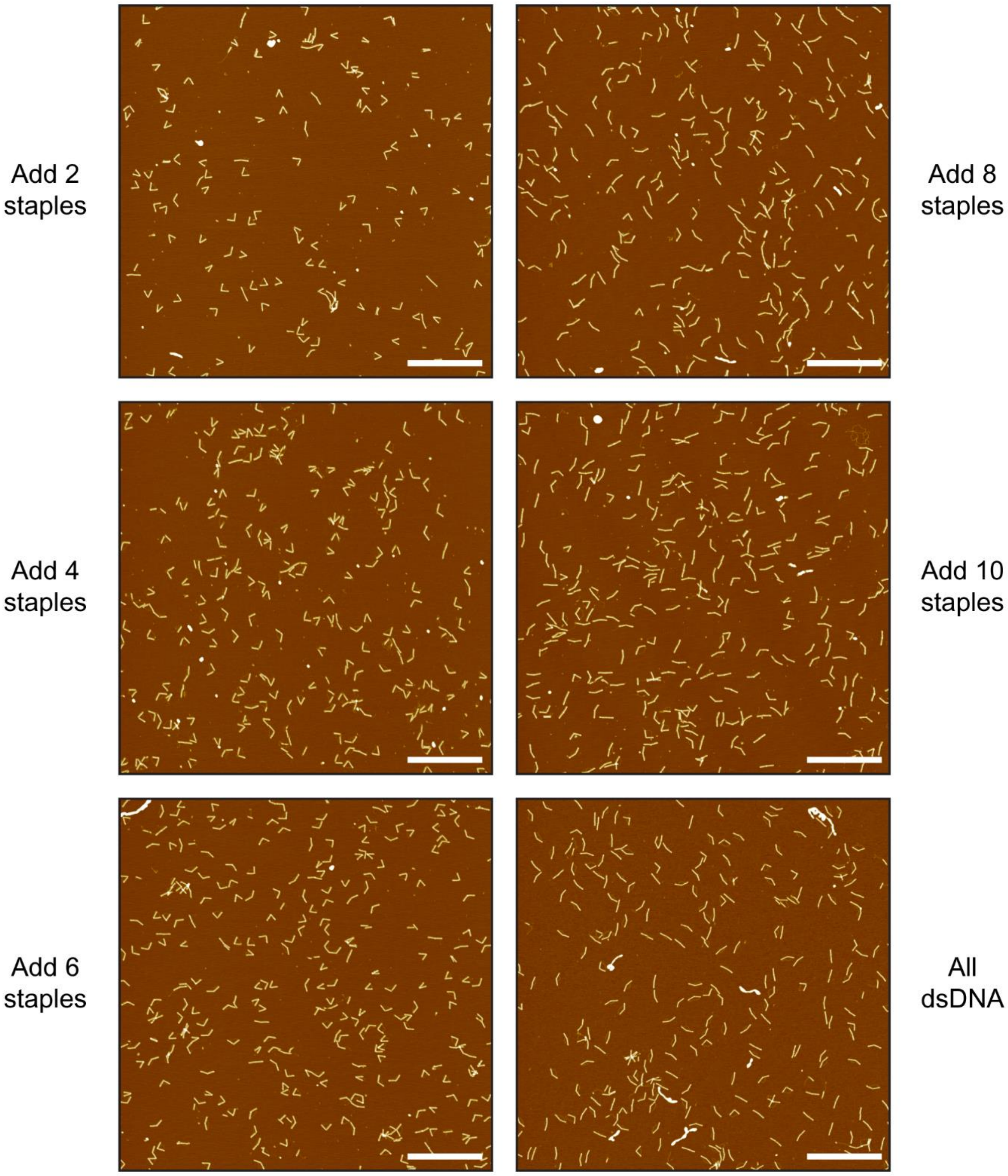

Supplementary Figure 45. AFM images of the structures measured and analyzed in Supplementary Figs. 43 and 44. Scale bars: 1 μm.

**Left section pool**												
**L1 (17)**	L1_01	L1_02	L1_03	L1_04	L1_05	L1_06	L1_07	L1_08	L1_09	L1_10	L1_11	L1_12
	L1_ds1	L1_ds2	L1_ds3	L1_ss4	L1_ss5							
**L2 (10)**	L2_01	L2_02	L2_03	L2_04	L2_05	L2_06	L2_07	L2_ss1	L2_ss2	L2_ss3		
**L3 (11)**	L3_01	L3_02	L3_03	L3_04	L3_05	L3_06	L3_07	L3_08	L3_ss1	L3_ss2	L3_ss3	
**Middle section pool**												
**M1 (10)**	M1_01	M1_02	M1_03	M1_04	M1_05	M1_06	M1_07	M1_ss1	M1_ss2	M1_ss3		
**M2 (11)**	M2_01	M2_02	M2_03	M2_04	M2_05	M2_06	M2_07	M2_08	M2_ss1	M2_ss2	M2_ss3	
**M3 (10)**	M3_01	M3_02	M3_03	M3_04	M3_05	M3_06	M3_07	M3_ss1	M3_ss2	M3_ss3		
**Right section pool**												
**R1 (11)**	R1_01	R1_02	R1_03	R1_04	R1_05	R1_06	R1_07	R1_08	R1_ss1	R1_ss2	R1_ss3	
**R2 (10)**	R2_01	R2_02	R2_03	R2_04	R2_05	R2_06	R2_07	R2_ss1	R2_ss2	R2_ss3		
**R3_504 (18)**	R3_01	R3_02	R3_03	R3_04	R3_05	R3_06	R3_07	R3_08	R3_09	R3_10	R3_11	
	R3_504_1	R3_504_2	R3_504_3	R3_504_4	R3_504_5	R3_504_6	R3_504_7					
**Seam section pool**												
**A1 (7)**	A1_01	A1_02	A1_03	A1_04	A1_ss1	A1_ss2	A1_ss3					
**A2 (8)**	A2_01	A2_02	A2_03	A2_04	A2_05	A2_ss1	A2_ss2	A2_ss3				
**A3 (7)**	A3_01	A3_02	A3_03	A3_04	A3_ss1	A3_ss2	A3_ss3					
**A4 (8)**	A4_01	A4_02	A4_03	A4_04	A4_05	A4_ss1	A4_ss2	A4_ss3				
**A5 (7)**	A5_01	A5_02	A5_03	A5_04	A5_ss1	A5_ss2	A5_ss3					
**A6 (8)**	A6_01	A6_02	A6_03	A6_04	A6_05	A6_ss1	A6_ss2	A6_ss3				
**A7 (7)**	A7_01	A7_02	A7_03	A7_04	A7_ss1	A7_ss2	A7_ss3					
**A8 (8)**	A8_01	A8_02	A8_03	A8_04	A8_05	A8_ss1	A8_ss2	A8_ss3				
**dsDNA adjuster strut staples**												
**ADJ (12)**	ADJ_01	ADJ_02	ADJ_03	ADJ_04	ADJ_05	ADJ_06	ADJ_07	ADJ_08	ADJ_09	ADJ_10	ADJ_11	ADJ_12

Supplementary Table 1. Reference staple set. Based on the reference staples here, we can easily constitute the staple sets for structural variations. See Supplementary Data for detailed staple set of each design and the sequence of all staple strands.

Structures shown in Fig. 2												
	#1	#2	#3	#4	#5	#6	#7	#8	#9	#10	#11	#12
**Eliminated staples**	18	19	20	22	19	23	20	26	35	37	33	35
**New staples**	7	7	7	7	7	7	7	7	7	7	7	7
**Total staples**	169	168	167	165	168	164	167	161	152	150	154	152
**Staple change**	3.9%	3.9%	3.9%	3.9%	3.9%	3.9%	3.9%	3.9%	3.9%	3.9%	3.9%	3.9%
	#13	#14	#15	#16	#17	#18	#19	#20	#21	#22	#23	#24
**Eliminated staples**	46	48	30	44	44	44	50	53	61	44	55	40
**New staples**	9	9	7	11	20	23	19	19	19	10	14	23
**Total staples**	143	141	157	147	156	159	149	146	138	146	139	163
**Staple change**	5.0%	5.0%	3.9%	6.1%	11.1%	12.8%	10.6%	10.6%	10.6%	5.6%	7.8%	12.8%
**Structures shown in Fig. 3**												
	0°	15°	30°	45°	60°	75°	90°	105°	120°	135°	150°	180° (ref)
**Eliminated staples**	30	28	27	26	24	23	22	21	20	19	19	0
**New staples**	7	9	10	10	9	10	10	10	10	9	10	0
**Total staples**	157	161	163	164	165	167	168	169	170	170	171	180
**Staple change**	3.9%	5.0%	5.6%	5.6%	5.0%	5.6%	5.6%	5.6%	5.6%	5.0%	5.6%	0%

Supplementary Table 2. The number of eliminated and replaced staples compared with the reference structure. The amount of staple change is defined as the ratio of the staples having different sequences from those of the reference staple set (the number of new staples divided by the number of reference staples).

**Structure**	Ref	#1	#2	#3	#4	#5	#6	#7	#8
**Monomer folding yield**	82.7%	77.2%	80.2%	78.0%	76.2%	77.0%	75.4%	78.2%	77.4%
**Structure**	#9	#10	#11	#12	#13	#14	#15	#16	
**Monomer folding yield**	79.7%	73.5%	82.8%	81.7%	82.2%	78.8%	82.1%	78.5%	
**Structure**	#17	#18	#19	#20	#21	#22	#23	#24	
**Monomer folding yield**	81.6%	80.7%	85.0%	84.4%	86.3%	89.3%	87.3%	89.6%	

Supplementary Table 3. Monomer folding yield of all structures calculated from gel electrophoresis in Supplementary Fig. 17. The monomer folding yield of each structure was calculated as the intensity ratio between the leading monomer band and all bands.

**Structure**	Ref	#1	#2	#3	#4	#5	#6	#7	#8
**Num of sample**	281	305	408	302	338	258	467	364	410
**Num of well-folded structures**	273	263	342	279	262	241	410	336	380
**Structural folding yield**	97.2%	86.2%	83.8%	92.4%	77.5%	93.4%	87.8%	92.3%	92.7%
**Structure**	#9	#10	#11	#12	#13	#14	#15	#16	
**Num of sample**	497	327	382	432	443	397	370	485	
**Num of well-folded structures**	313	233	208	245	190	184	155	158	
**Structural folding yield**	63.0%	71.3%	54.5%	56.7%	42.9%	46.3%	41.9%	32.6%	
**Structure**	#17	#18	#19	#20	#21	#22	#23	#24	
**Num of sample**	471	257	270	262	325	352	274	433	
**Num of well-folded structures**	378	120	131	186	173	296	110	254	
**Structural folding yield**	80.3%	46.7%	48.5%	71.0%	53.2%	84.1%	40.1%	58.7%	

Supplementary Table 4. Structural folding yield result of all 24 structures shown in Fig. 2. Structural folding yield is defined as the number of well-folded structures divided by the number of samples.

**Target**	15°	30°	45°	60°	75°	90°	105°	120°	135°	150°
**Num of samples**	517	576	606	776	623	715	727	637	708	530
**Average**	14.0°	29.3°	42.4°	59.0°	74.5°	88.4°	103.0°	119.1°	130.2°	143.8°
**Stdev**	3.3°	4.7°	5.6°	5.3°	8.5°	8.6°	7.7°	10.9°	11.5°	16.3°
**R^2 (Gaussian)**	0.951	0.967	0.985	0.993	0.972	0.985	0.975	0.955	0.921	0.833

Supplementary Table 5. Detailed experimental data of measured included angles shown in Fig. 3.

Supplementary References

1. Kim, D.-N., Kilchherr, F., Dietz, H. & Bathe, M. Quantitative prediction of 3d solution shape and flexibility of nucleic acid nanostructures. *Nucleic Acids Res.* **40,** 2862-2868 (2012).
2. Marko, J. F. & Siggia, E. D. Stretching DNA. *Macromolecules* **28,** 8759-8770 (1995).
3. Brooks, B. R., Janežič, D. & Karplus, M. Harmonic analysis of large systems. I. Methodology. *J. Comput. Chem.* **16,** 1522-1542 (1995).
4. Thomson, W. *Theory of vibration with applications* (CRC Press, 1996).
5. Douglas, S. M. *et al.* Rapid prototyping of 3d DNA-origami shapes with cadnano. *Nucleic Acids Res.* **37,** 5001-5006 (2009).
