# Supplementary material for: Polymorphic design of DNA origami structures through mechanical control of modular components
Source: Nat Commun. 2017 Dec 12;8:2067. doi: 10.1038/s41467-017-02127-6 (PMC5727162; doi:10.1038/s41467-017-02127-6)
Supplement: Supplementary file 3 — Description of Additional Supplementary Files [file 41467_2017_2127_MOESM3_ESM.pdf]

Description of Additional Supplementary Files

File Name: Supplementary Data 1
Description: Data for detailed staple set of each design and the sequence of all strands
